# Relationship between Freezing of Gait and Anxiety in Parkinson's Disease Patients: A Systemic Literature Review

**DOI:** 10.1155/2019/6836082

**Published:** 2019-07-24

**Authors:** Ivan Witt, Hooman Ganjavi, Penny MacDonald

**Affiliations:** ^1^The Brain and Mind Institute, The University of Western Ontario, London, Ontario, Canada; ^2^Department of Psychiatry, The University of Western Ontario, London, Ontario, Canada; ^3^Department of Clinical Neurological Sciences, The University of Western Ontario, London, Ontario, Canada

## Abstract

Freezing of gait (FOG) is experienced by a significant number of patients with Parkinson's disease (PD). The pathophysiology of this disabling motor symptom remains unclear, and there are no effective therapies. Anxiety has previously been posited as a contributing factor to gait freezing. There have been few studies directly investigating this topic, and a comprehensive literature review is lacking. The objective of this paper was to systematically review the evidence associating anxiety with the presence, severity, and progression of FOG in PD patients. The PubMed, EMBASE, and PsycINFO databases were searched up to September 19, 2018, for English-language, peer-reviewed articles that explored anxiety and FOG as outcome measures in a PD population base. Review articles, case reports, and articles that assessed gait disorders other than FOG were excluded, yielding a total of 26 articles in the final analysis. Of these 26 studies, 16 had a significant relationship between anxiety outcome measure and either presence or severity of FOG. There was great variability among studies in terms of outcome measures for both FOG and anxiety. Despite this heterogeneity, most studies relate anxiety and FOG. Standardized, high-validity outcome measures of anxiety and FOG are needed. Future exploration should aim to clarify the role of anxiety in FOG as a causal factor, pathophysiological marker, and manifestation of a common pathophysiological process versus a consequence of FOG itself. Clarifying the relationship between anxiety and FOG could reveal anxiety reduction as a therapy for FOG.

## 1. Introduction

Parkinson's disease (PD) is a neurological movement disorder characterized by four cardinal motor symptoms: bradykinesia, rigidity, resting tremor, and postural instability [1]. It is a progressive disease with no cure and no current disease-modifying therapies [2], and 60–80% of dopamine-producing neurons in the substantia nigra pars compacta degenerate over the course of the disease, leading to substantial dopamine reduction in the dorsal striatum and the aforementioned motor symptomatology [3]. These motor symptoms progress in severity, eventually leading to diminished ability to perform basic activities of daily living. Pharmacological dopamine replacement therapy is used to mitigate disabling PD motor sequelae, helping preserve quality of life in the earlier stages of PD, but ultimately produces disabling side effects at higher doses which renders this treatment undesirable in the later stages of PD [4]. PD is further characterized by additional motor (i.e., gait freezing, poor balance, and speech disturbances) and nonmotor (i.e., depression, anxiety, sleep disturbances, and fatigue) symptoms [5].

Freezing of gait (FOG) is a particularly debilitating motor symptom experienced by 50–80% of PD patients [6, 7]. The third item on the Freezing of Gait (FOG) Questionnaire reads as follows: “Do you feel that your feet get glued to the floor while walking, making a turn, or trying to initiate walking (i.e., freezing)?” [8]. This item functions as a reliable screening tool for the presence of FOG. Intermittent in nature, experienced at later disease stages by 47% of PD patients at least bimonthly [6], episodes of FOG increase susceptibility to falls, limit mobility, and reduce quality of life [9]. Much remains to be learned regarding the pathogenesis of FOG. A small but growing body of research indicates several variables likely contribute to this phenomenon, including PD motor phenotype, cognitive load, emotional stimuli, and environmental factors [9].

Anxiety is a common, nonmotor symptom of PD, experienced by anywhere from 40% to 69% of Parkinson's patients [10, 32]. PD is often preceded by a prodromal state of anxiety, with presence of anxiety positively associated with intensity of motor impairments in PD [10]. Previous studies have examined the interrelationship between anxiety and motor symptomatology in PD, but the link between anxiety and FOG in PD is not well characterized.

The aim of this literature review is to explore the degree to which anxiety in PD is associated with eventual development and/or severity of FOG specifically. Exploring scientific consensus regarding the relation between anxiety disorders and FOG in PD might reveal insights into the pathophysiology of FOG and may suggest treatment options for FOG not previously explored. As no current literature review exists for this topic, this paper serves as a benchmark in framing the current scientific understanding of anxiety's relationship to FOG, thereby facilitating future research directions and treatment discoveries.

## 2. Methods

A systematic review of the PsycINFO, EMBASE, and PubMed databases was performed to uncover studies published before September 19, 2018, in which both anxiety and FOG in PD were quantified and compared. To generate an initial list of articles, the following search criteria were applied to each database: (“Parkinson's disease” OR “Parkinson disease” OR “Parkinson's) AND (“Anxiety” OR “Generalized Anxiety Disorder” OR “Panic Disorder” OR “Anxiety Disorder” OR “Panic Attack”) AND (“Freezing of Gait” OR “Gait Freezing”).

**Figure 1:**
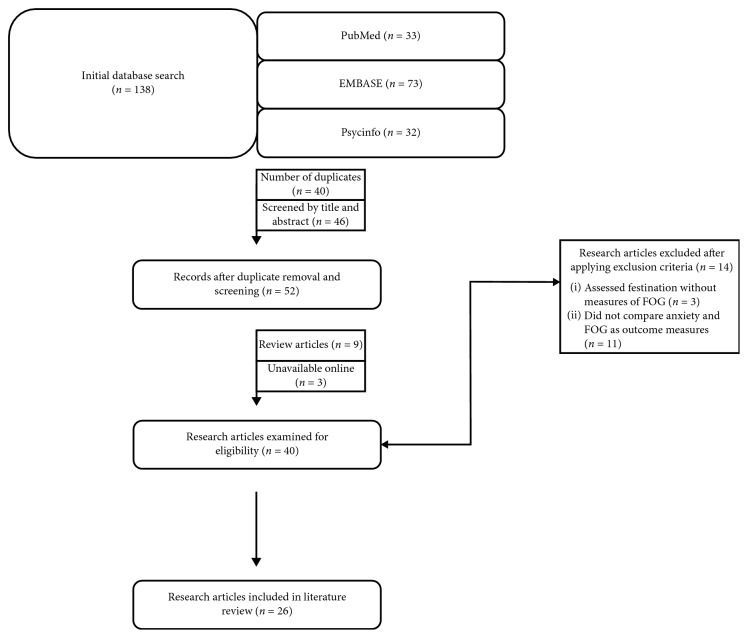


A total of 138 articles were found from the initial database search: 32 on PsycINFO, 73 on EMBASE, and 33 on PubMed. English-language, peer-reviewed studies investigating factors of anxiety and FOG in patients with a confirmed diagnosis of PD were included in this review. Articles were excluded if they did not document and compare anxiety and FOG as outcome measures, or if they assessed festination/other gait disorders only and did not clearly also provide measures of FOG. Additionally, reviews, case report articles, and conference abstracts were excluded from this analysis. See Table 1 for the list of inclusion and exclusion criteria. The PRISMA Statement was used to guide the systematic literature review.  Figure 1 shows a flow chart of the study retrieval process.

## 3. Results

Our search revealed studies that were quite heterogeneous in terms of demographics, anxiety measures, and FOG measures. The details and findings of each study are summarized in Table 2.

### 3.1. Demographics

Our analysis examines research from 11 countries with a combined total of 3,963 PD patients. All studies included were conducted within the last 12 years, with 20 of the 26 published between 2014 and 2018. This recent influx of research speaks to the increased awareness of the relation between anxiety and FOG in PD. Nine of the included studies used age-matched controls: [24, 30, 31, 36, 37, 39–41, 43].

### 3.2. Measures of Parkinson's Disease

Participants in all 26 studies had a confirmed diagnosis of PD by a licensed neurologist. The severity of PD was estimated with the Unified Parkinson's Disease Rating Scale-Motor Subscale (UPDRS-III) for all patients in each of the studies included in this review.

### 3.3. Measures of Anxiety

#### 3.3.1. Hospital Anxiety and Depression Scale-Anxiety Component (HADS-A)

Eleven studies employed the HADS-A to quantify anxiety levels. The HADS is a 14-item self-assessment scale developed to detect depression and anxiety in the hospital setting [11]. The anxiety component consists of 7 items with scores ranging from 0 to 3, with 0 being the lowest anxiety level and 3 being the highest, for a range in score of 0–21.

#### 3.3.2. Hamilton Anxiety Rating Scale (HAMA)

Eight studies used the HAMA as an outcome measure for anxiety. The HAMA was designed as a clinician-based questionnaire but is also used as a self-assessment scale. It consists of 14 items related to symptoms and signs of anxiety graded on a 5-point scale from 0 to 4 (with 0 being the least severe and 4 being the most severe) with a range in score of 0–56 [12]. Items 10 and 11 of the Hamilton Depression Rating Scale are anxiety related (HAMD-A), with both items scored on a 0–4 scale for a total HAMD-A component score of 8 [13]. Patients in the study of Zhang et al. [18] and Yao et al. [24] completed the HAMD-A in addition to the HAMA.

#### 3.3.3. Beck Anxiety Inventory (BAI)

Four studies employed the BAI as an outcome measure for anxiety. The BAI is a 21-item 4-point self-report scale developed as a measure to discriminate between depressive and anxiety symptoms [14]. Items are scored from 0 (least anxiety severity) to 3 (most anxiety severity) with a score range from 0 to 63.

#### 3.3.4. State and Trait Anxiety Inventory Scale (STAT-I)

One study implemented the STAT-I as an outcome measure for anxiety. The STAT-I is a 40-item 4-point self-report scale comprising 2 categories: state anxiety (20 items) and trait anxiety (20 items). Items are scored as follows: 1 = almost never, 2 = somewhat, 3 = moderately, and 4 = almost always, with cumulative scores ranging from 20 to 80 [15].

#### 3.3.5. Parkinson's Disease Questionnaire (PDQ-39)

Only 1 study used a subset of the PDQ-39 as an outcome measure for anxiety, specifically Items 21 and 9 which read as follows: “During the last month have you felt anxious?” and “During the last month have you felt frightened or worried about falling over in public?” [16]. Scores on these items ranged from 0 to 4 (0 = never, 1 = occasionally, 2 = sometimes, 3 = often, and 4 = always), with Item 21 used as a general measure of anxiety and Item 9 used as a subjective measure of anxiety during walking [42].

#### 3.3.6. Parkinson's Anxiety Scale (PAS)

Only 1 study utilized the PAS as an outcome measure for anxiety (*n* = 1). The PAS is a 12-item observer- or self-reported scale that is composed of 3 subscales: persisting anxiety (5 items), episodic anxiety (4 items), and avoidance behaviour (3 items). The PAS is scored on a scale of 0–4, with 0 representing “not or never” and 4 representing “severe or almost always,” and the score range is from 0 to 48 [29].

### 3.4. Measures of FOG

The studies included in the review are particularly heterogeneous in their approach to assessing FOG. Various combinations of the Freezing of Gait Questionnaire (FOG-Q), convincing subjective reports of the FOG phenomenon and/or direct observation of FOG by an experienced clinician/researcher, were used across studies (with the exception of [22, 23, 32, 34], which used Item 14 of the UPDRS-II as their measure of self-reported FOG). The entire FOG-Q is a 6-item questionnaire with 4 questions specific to FOG severity and 2 questions about general gait difficulties, with responses graded on a 5-point scale from 0 to 4 [8]. The 3rd item of this questionnaire (Do you feel that your feet get glued to the floor while walking, making a turn, or trying to initiate walking (i.e., freezing)?) is often used alone, with a score ≥1 meeting criteria for FOG in several studies.

Six studies implemented a new version of the Freezing of Gait Questionnaire (nFOG-Q) that was designed by Nieuwboer et al. [17] to better assess FOG severity in PD patients which was largely based on the original FOG-Q. The nFOG-Q adds an additional item to the beginning of the FOG-Q that “allows FOG detection and exclusion of patients without the symptom from the actual scoring of FOG severity and impact” [17] and a video presentation demonstrating variants of FOG for patients/caregivers. Ehgoetz Martens et al. [29] implemented a newly designed Characterizing Freezing of Gait questionnaire (C-FOG)—a 35-item self-reported scale divided into 4 sections: severity, triggers, strategies (to reduce or overcome FOG), and other types of freezing (i.e., upper limb freezing) [29]. Based on the C-FOG responses, PD patients with FOG are further divided into asymmetric motor, anxious, and sensory-attention freezing subtypes.

Objective measures of FOG were also collected during some experimental paradigms through either virtual reality paradigms, gait tasks, positional sensors, or some combinations thereof.

### 3.5. Positive Studies

Sixteen of the 26 studies demonstrated a significant relationship between their respective anxiety outcome measure and FOG presence and/or severity, therefore meeting criteria for a positive result. However, the studies differ extensively in their methodology including different anxiety measures, study design, and FOG quantification. Their details are given below.

#### 3.5.1. Zhang et al. [18]: A Three-Year Prospective Longitudinal Study in a Chinese Population

There was no significant association between the total HAMA, HAMA Somatic Anxiety, and HAMA Psychic Anxiety baseline scores and development of FOG 3 years after study. In contrast, the HAMD Anxiety/Somatic score was significantly higher in PD patients with FOG than that in PD patients without FOG 3 years after study. Further binary logistic regression revealed that the HAMD Anxiety/Somatic score was significantly associated with FOG development 2 years later, but not 1 year later or 3 years later. Ultimately, the study concluded no significant associations between HAMA scores and FOG but discovered higher scores on the HAMD Anxiety/Somatic subscale were significantly associated with the presence of FOG 2 years later. Risk factors for the development of FOG changed yearly in this patient population and comprised a combination of nonmotor and motor symptomatology.

#### 3.5.2. Ehgoetz Martens et al. [19]: A Prospective Longitudinal Study Conducted on a Larger Cohort of Cases in an Australian Population

The mean HADS-A score in PD + FOG was significantly higher than that in PD-NF, with a significant main effect of group observed. This group effect remained significant with additional covariate analysis using the UPDRS-III score, mean PD duration, and MMSE score as covariates. In contrast, the mean HADS-A score in PD + mild FOG was not significantly different from that in PD + FOG. A significant correlation between the severity of anxiety symptoms and FOG-Q3 scores emerged across all groups.

Further analysis to assess the predictive value of the HADS-A score for the presence of FOG in PD involved merging the original PD + mild FOG and PD + FOG groups into one single PD + FOG group, with the original PD-NF group remaining intact during comparison. This final analysis revealed a positive predictive value of 28.26% for identifying patients with FOG and a negative predictive value of 89.61% for identifying patients without FOG. This study concluded a significant relationship between anxiety and presence of FOG and implicated anxiety as a potential biomarker for FOG development in PD patients.

#### 3.5.3. Walton et al. [20]: A Cross-Sectional Analysis of Baseline Assessment Data from the University of Sydney's Parkinson's Disease Research Clinic

HADS-A scores were significantly correlated with FOG severity. The study's main conclusion centred on self-reported FOG as a significant independent contributor to quality of life in PD and evidenced a statistically significant relationship between anxiety and FOG severity through self-reported measures.

#### 3.5.4. Ou et al. [21]: An Observational Cross-Sectional Study in a Chinese Population to Explore the Prevalence and Clinical Correlates of FOG

In analysis of covariance adjusting for age, disease duration, and H&Y stage, the total mean score on the HAMA for freezers was significantly higher than that for nonfreezers. A binary logistic regression analysis indicated no significant correlation between the HAMA anxiety score and FOG. Notably, age- and disease duration-adjusted ANOVA indicated that the freezers had lower total scores on the MMSE and Montreal Cognitive Assessment compared to nonfreezers, and lower MMSE scores were not excluded from the final analysis. The study ultimately concluded that while mean HAMA anxiety scores were significantly higher in freezers than those in nonfreezers, HAMA was not a significant clinical correlate related to FOG.

#### 3.5.5. Burn et al. [22]: An Observational Cross-Sectional Study in a UK Population That Sought to Identify Associations between Phenotypic Variants of Motor and Mood Subtypes in PD

The mean total HADS-A score in freezers was significantly higher than that in nonfreezers, with a significantly higher proportion of freezers meeting the HADS-A ≥ 11 criteria for anxiety compared to nonfreezers (20%). Follow-up logistic regression analysis revealed that an HADS-A score ≥11 (Class 3) was not significantly predicted by FOG status.

#### 3.5.6. Lieberman [23]: An Observational Cross-Sectional Study in a US Population to Study the Association between Anxiety, Depression, and Panic Attacks in PD Patients with FOG

Freezers in this study population had significantly higher HAMA scores compared to nonfreezers. Additionally, panic attacks were significantly increased in freezers compared to nonfreezers. Ultimately, FOG presence in PD was significantly associated with anxiety and panic attacks in this study.

#### 3.5.7. Yao et al. [24]: A Case-Control Study in a Chinese Population Investigating Whether Cognitive Impairment Was Correlated with FOG in PD Patients

After adjusting for age, no significant differences were found between HAMA total, HAMA Somatic Anxiety, and HAMA Psychic Anxiety between freezers and nonfreezers, but the HAMD Anxiety/Somatic score was significantly higher in freezers than that in nonfreezers. Spearman's rho correlation between HAMD-A and nFOG-Q demonstrated severity of FOG positively correlated with higher anxiety subscale scores within the FOG subgroup. Though the study indicated no significant relationship between HAMA and FOG in PD, the HAMD Anxiety/Somatic score was significantly related to FOG status.

#### 3.5.8. Rahimi et al. [25]: A Prospective, Experimental, Pre/Posttreatment Study in a Canadian Population Wherein Optimally Medicated PD Patients with Intractable FOG Were Treated with a 90-Day Course of 1 mg of Rasagiline per Day, with FOG Frequency/Duration and Cognitive/Motor Measures Assessed prior to and following Treatment

A hierarchical clustering analysis was utilized to identify subgroups of participants with similar responses to rasagiline therapy based on FOG frequency and duration. Subsequently, participants were divided into the following three groups following treatment with rasagiline: improved (*n* = 6), worsened (*n* = 5), and no change (*n* = 3). There were no significant differences in pre/posttreatment BAI scores, or overall count and duration of FOG episodes. The high variability in individual scale scores led to null effects across measures.

As the no-change group did not exhibit any episodes of FOG, they were excluded from follow-up bidirectional elimination-stepwise regression analysis (thereby avoiding floor effects) to identify the clinical variables which predicted reduction in frequency of FOG events after treatment. Higher BAI scores proved to be a statistically significant predictor of FOG worsening following rasagiline therapy, and the final predictive model explained 99% of the variance between the improved and worsened groups as a function of treatment with rasagiline.

#### 3.5.9. Ehgoetz Martens et al. [26]: A Longitudinal Study Conducted in an Australian Population

HADS-A scores increased significantly from baseline to follow-up across all 3 groups, and both continuing and transitional freezers had significantly greater mean HADS-A scores compared to nonfreezers at baseline and follow-up. Correlational analysis revealed HADS-A scores were significantly associated with FOG-Q total scores.

A predictive model was constructed with FOG-Q total and HADS-A among the 7 predictive variables included, the overall predictive success of which was 84% (correctly classified 69.6% of transitional freezers and 90.4% of nonfreezers). Since FOG-Q total and HADS-A were the strongest predictors identified in this model, a subsequent model was constructed that only included these two variables to enhance clinical relevance. This final model was able to correctly identify 64.9% of patients who developed FOG and 90% of patients who remained nonfreezers with an overall predictive success of 82.1%. The study concluded that anxiety was a crucial variable to consider in accurately predicting the onset of FOG in PD patients and suggested HADS-A scores may be a useful screening tool for clinicians to identify patients at risk for FOG development.

#### 3.5.10. Ou et al. [27]: A Longitudinal Study in a Chinese Population to Evaluate Clinical Predictors of FOG

In this sample, significantly higher mean HAMA scores at baseline and follow-up were observed in freezers compared to nonfreezers; however, the annual change in the HAMA score did not differ significantly between the two groups. Ultimately, the HAMA score was not determined to be a significant predictor of FOG development in this cohort, though HAMA scores were higher in freezers.

#### 3.5.11. Ehgoetz Martens et al. [28]: An Experimental Task-Based Functional MRI Study in an Australian Population to Investigate the Neural Heterogeneity of Freezing of Gait

Higher degrees of functional connectivity between the limbic network and the ventral striatum as well as the cognitive control network (comprising the anterior cingulate cortex, dorsolateral prefrontal cortex, and posterior parietal cortex) during freezing episodes were observed compared to normal foot tapping. Higher HADS scores were associated with this increased coupling. Anticoupling among the cortical and subcortical regions of the limbic network (and within the subcortical limbic region itself) was also noted during freezing relative to normal foot tapping, and this degree of anticoupling was also associated with a higher HADS score. Subcortical regions of the limbic network demonstrated tight coupling to the dorsal caudate (whereas the cognitive control network and the dorsal caudate nucleus were anticoupled). Lower HADS scores corresponded with less functional connectivity between the cortex and striatum. Ultimately, the study concluded that three unique phenotypes of gait freezing exist (cognitive, motor, and limbic), with functional neuroimaging of the limbic corticostriatal pathway suggesting a distinct limbic phenotype of FOG that was related to HADS scores.

#### 3.5.12. Ehgoetz Martens et al. [29]: An Experimental Study Performed in an Australian Population to Characterize Subtypes of Freezing of Gait with a Newly Generated FOG Questionnaire (C-FOG)

Higher mean PAS scores were observed in the anxious subgroup compared to the asymmetric-motor and sensory-attention subgroups. The study concluded that distinct freezing phenotypes could be identified using the C-FOG, with significantly higher PAI scores characterizing the anxiety subgroup.

#### 3.5.13. Vandenbossche et al. [30]: A Belgian Study Investigating Executive Functioning and Attention in PD Patients with FOG

The HADS-A was significantly associated with nFOG-Q total scores. The study concluded that an impairment in conflict resolution was significantly associated with FOG in PD and evidenced a significant correlation between FOG severity and HADS-A scores.

#### 3.5.14. Kostic et al. [31]: An MRI Study in a Serbian Population to Explore Patterns of Grey Matter (GM) Tissue Loss in PD Patients with FOG

Mean HAMA scores were higher in PD-FOG than those in PD-noFOG and healthy controls. Ultimately, the study discovered a pattern of GM atrophy in the frontal and parietal cortices associated with FOG presence and reported significantly higher HAMA scores in PD patients with FOG.

#### 3.5.15. Ehgoetz Martens et al. [32]: A Canadian Study Where Anxiety Was Induced via a Virtual Reality Apparatus and the Presence/Severity of FOG Subsequently Measured in PD Patients Both on and off Dopaminergic Medications

The primary outcome measure that quantified FOG was the percent of each trial frozen, with freezers spending a significantly greater portion of time frozen in the high condition compared to the low condition. In addition, freezers exhibited significantly higher numbers of freezing episodes in the high condition relative to the low condition, but no significant differences in the duration of freezing. Concomitantly, freezers self-reported significantly higher levels of anxiety during the experiment compared to nonfreezers, with both groups reporting significantly higher levels of anxiety in the high condition compared to the low condition. Anxiety severity was significantly higher when freezers were tested off their dopaminergic medications compared to on medications, and none of the FOG variables were statistically compared between medication states secondary to the limited dataset and lack of variance.

This study concluded that anxiety plays a causal role in FOG. By manipulating anxiety-inducing condition via virtual reality, Ehgoetz Martens et al. [32] were able to elicit over 300 freezing episodes in 85.7% of PD patients with FOG—a feat previously unheard of in the literature. The high condition resulted in a significantly higher number of FOG episodes and percentage of time frozen compared to the low condition in the freezing population, with freezers reporting significantly higher levels of anxiety compared to nonfreezers. Neural correlates were inferred based on these findings in conjunction with prior neuroimaging studies of FOG patients, which implicated disrupted emotive processing in the ventral striatum and anxiety-induced limbic overactivity as potential mechanisms underlying FOG.

#### 3.5.16. Pimenta et al. [33]: A Cross-Sectional Study in a Brazilian Population Examining the Relationship between Anxiety and FOG in PD

HADS-A scores were significantly higher in freezers than those in nonfreezers, and HADS-A scores were significantly correlated with both FOG-Q3 and FOG severity. HADS-A scores ≥8 were determined to accounting for 38% of the variance in FOG severity scores in this study population. The study concluded that the HADS-A was an independent contributor to FOG severity and recommended routine anxiety evaluations in PD patients presenting with FOG.

### 3.6. Negative Studies

Ten of the 26 studies evidenced no significant relationship between their respective anxiety outcome measure and FOG presence and/or severity, therefore meeting criteria for a negative result. Once again, the studies differ extensively in their methodology including different anxiety measures, study design, and FOG quantification. Their details are given below.

#### 3.6.1. Perez-Lloret et al. [34]: An Observational Cross-Sectional Study Conducted in France to Determine the Prevalence of FOG in a Sample of PD Patients and Assess Its Relation to Quality of Life-Related Clinical Correlates (including Anxiety)

No significant differences in HADS-A scores between freezers and nonfreezers were found. The study ultimately concluded that FOG in PD patients correlates with reduced quality of life, disease severity, cognitive deficit, and exposure to antimuscarinics, but no correlation was found with presence of anxiety in this study population.

#### 3.6.2. Hall et al. [35]: A Cross-Sectional Analysis of Baseline Assessment Data Gathered from a Larger Cohort of Cases at the University of Sydney's Parkinson's Disease Research Clinic from 2008 to 2015

Though the mean HADS-A score was higher in freezers than that in nonfreezers, this difference did not prove to be statistically significant following Mann–Whitney's *U*-test. The study concluded early-stage PD patients with FOG had increased nontremor motor features (speech, swallowing, facial expression difficulties, etc.) and selective impairments of working memory/attentional set-shifting, but not significantly increased anxiety levels compared to nonfreezers in this study population.

#### 3.6.3. Huh et al. [36]: A Case-Control Cross-Sectional Experiment in a South Korean Population Examining Postural Sensory Deficits Linked to FOG Severity through a Computerized Dynamic Posturography System

PD patients with FOG and PD patients without FOG had significantly higher BAI scores compared to controls with no significant differences between groups. The authors concluded that specific postural sensory deficits (i.e., disrupted somatosensory feedback and vestibular input integration) were associated with FOG in PD, but BAI scores were not.

#### 3.6.4. Raffo De Ferrari et al. [37]: An Experimental Study Conducted in an Italian Population Examining the Relationship between Theory of Mind and FOG Presence in PD Patients

No significant main effect of group was found for BAI scores. The study concluded that affective theory of mind was impaired in the PD-FOG+ group compared to the PD-FOG*−* group, but no significant differences for self-reported anxiety were apparent.

#### 3.6.5. Rubino et al. [38]: An Italian Study Examining Differences in Grey Matter Volume (GM) between PD Patients with and without FOG

The mean HAMA score was higher in the FOG*−* group than that in the FOG+ group, but this difference was not statistically significant. The study localized the posterior parietal cortex as a site of cortical volume reduction in FOG+ participants relative to the FOG*−* group, but these groups did not differ significantly in their HAMA scores.

#### 3.6.6. Vandenbossche et al. [39]: A Belgian Study That Investigated Whether Sequence Learning Was Diminished in PD Patients with FOG, with Participants Completing a Serial Reaction Time Task with Random or Sequenced Block Conditions

Though the mean HADS-A score was higher in freezers than that in nonfreezers and controls, an independent *t*-test did not yield any statistically significant differences between these groups. The study discovered significant impairments in automaticity in PD patients with FOG, but no significant differences or association between anxiety levels and FOG in PD patients.

#### 3.6.7. Heremans et al. [40]: A Study Conducted in a Belgian Population to Explore Handwriting Differences in PD Patients with FOG

The mean HADS-A score was higher in freezers than that in nonfreezers, but this difference was not statistically significant. The study discovered writing was more severely negatively impacted in PD patients with FOG compared to PD patients without FOG, and the HADS-A scores did not differ significantly between these groups.

#### 3.6.8. Stefanova et al. [41]: An Experimental Study Performed in a Serbian Population to Explore Whether Attentional Set-Shifting and Inhibitory Control Were Associated with FOG in PD

HAMA differences between freezers and nonfreezers were not statistically significant. The study concluded specific attentional set-shifting dysfunctions were associated with FOG in PD, but no significant differences were apparent in anxiety measures between freezers, nonfreezers, or healthy age- and sex-matched controls.

#### 3.6.9. Gilat et al. [42]: A Resting-State MRI Study Investigating Functional Connectivity Differences in PD Patients with and without FOG in an American Population

Worsening severity of FOG was associated with greater degrees of anticoupling between the amygdala and regions of the frontoparietal attention control network. Item 21 of PDQ-39 was not significantly correlated with the functional connectivity scores in freezers and nonfreezers. A significantly negative association between Item 9 of the PDQ-39 and the functional connectivity between the bilateral amygdala and the frontoparietal attention control network was discovered in freezers, but not nonfreezers. No other statistically significant functional connectivity relationships emerged between Item 9 scores in freezers and nonfreezers. The study ultimately provided neuroimaging evidence for increased baseline striato-limbic connectivity in freezers compared to nonfreezers. The anxiety outcome measure (Item 21 of PDQ-39) was not significantly different in freezers vs. nonfreezers and did not correlate significantly with striato-limbic connectivity, with the study authors noting a thorough assessment of mood disturbance had not been performed and recommending further research to explore the underlying pathophysiology of anxiety-induced FOG.

#### 3.6.10. Lagravinese et al. [43]: An Experimental Study Performed in an Italian Population to Examine the Effect of Emotional Stimuli on Gait in PD Patients with and without FOG

Mean BAI scores were lower in the PD-FOG+ group than those in the PD-FOG*−* group and higher than those in the ELD group. These differences were not statistically significant. The study concluded that emotional valence of stimuli affected gait initiation in the PD-FOG+ group compared to PD-FOG– patients and healthy controls, but anxiety measures did not differ significantly between these groups.

## 4. Discussion

To our knowledge, this is the first systematic literature review to summarize research findings on the association between anxiety and freezing of gait in PD. Most studies revealed that higher ratings on anxiety subscales were significantly correlated with FOG presence in PD and/or severity, with 16 of the 26 studies indicating a relationship between anxiety outcome measure and presence or severity of FOG.

### 4.1. Design

Nine of the included studies used age-matched controls: [24, 30, 31, 36, 37, 39–41, 43]. Of these studies, only Vandenbossche et al. [30] and Kostic et al. [31] reported statistically significant differences between anxiety outcome measures and FOG severity. Apart from Yao et al. [24], the primary focus of these age-matched control group studies was not anxiety. This review lacks a consistent evidence base of research comparisons to age-matched controls, and a comparator control group with anxiety levels as the primary objective of the research is greatly needed in further research of this topic. Additionally, 46% of the reviewed studies were observational in nature (*n* = 12). Of these observational studies, nine found a significant relationship between FOG presence or severity and anxiety outcome measure in PD patients, but only four of these had a longitudinal design. More prospective longitudinal designs are needed to further examine the causative role of anxiety in FOG development.

### 4.2. Anxiety

Significant heterogeneity existed in terms of the anxiety subscales used. Conflicting reports exist regarding the validity of these subscales in PD. The HADS-A, while useful as a screening tool for anxiety, has been criticized for lack of specificity in distinguishing depressive and anxiety symptoms in PD [44]. A Rasch analysis performed by Forjaz et al. [45] determined that the HADS-A was not suitable for assessing anxiety in PD, and evidence from multiple review articles corroborates this finding [46, 47]. A systematic literature review of studies that performed a latent factor analysis of the HADS published between 2000 and 2010 suggested that both its underlying structure and ability to detect depression or anxiety were uncertain [48].

Given that the HADS-A was the most commonly implemented anxiety outcome measure in this literature review, the questions of its applicability to PD are particularly relevant. Six of the eleven studies that implemented the HADS-A in this literature review demonstrated a significant relationship between FOG and anxiety: [19, 20, 22, 26, 30, 33]. Ultimately, an inconsistent pattern emerged with a wide range of associations between HADS-A and FOG presence/severity. On the one hand, Ehgoetz Martens et al. [26] and Pimenta et al. [33] suggested that the HADS-A score could potentially be a screening tool for FOG development based on the high predictive power of the score in their sampled populations, while on the other hand, Perez-Lloret et al. [34] and Hall et al. [35] reported no significant HADS-A differences between freezers and nonfreezers in their cohorts. Furthermore, Perez-Lloret et al. [34] required a score ≥7 on the scale to meet study criteria for anxiety, Pimenta et al. [33] required a score ≥8, and Burn et al. [22] required a score ≥11. A more consistent consensus on specific HADS-A criteria for anxiety would facilitate direct comparisons between studies more effectively, and more research needs to investigate both its suitability for PD patients and its association with FOG.

These same points can be made in relation to the other anxiety scales used. While some literature recommends the BAI as a more comprehensive and valid assessment of anxiety symptoms in PD [46], others report unsatisfactory interitem correlation, convergent validity, and factorial structure [49]. It is also noted that the BAI's item content is weighted towards physical symptoms of panic attacks as opposed to generalized anxiety. While the BAI showed some promising utility in Rahimi et al.'s small sample in predicting FOG treatment success [25], the remaining three studies that incorporated the BAI did not demonstrate a significant relationship between anxiety and FOG presence, and the conflicting evidence of its validity in PD is reminiscent of the issues encountered with the HADS-A. The same can be said for the HAMA—the second most common anxiety scale used in our review. Leentjens et al. [44] determined that the HAMA possessed satisfactory interitem correlation, convergent validity, and factorial structure. However, the positive predictive value of 0.63 was deemed poor. HAMA has also been criticized for bias towards persistent symptoms of anxiety as opposed to episodic anxiety or avoidance behaviour [49]. In our literature review, a significant association between HAMA and gait freezing was reported in three of the eight papers that used it (Ou et al. [21], Lieberman [23], and Kostic et al. [31]).

Many of the studies that used the HAMA also used the HAMD—a scale designed to quantify depressive symptoms. While we did not include the total HAMD score as part of our analysis, the HAMD-A component of the scale was investigated and reported in our results. Much like the HAMA, a factor analysis of the HAMD in PD revealed an unsatisfactory factorial validity, questioning its ability to assess the anxiety domain of the subscale in PD [50]. An interesting finding emerged in both Zhang et al. [18] and Yao et al.'s [24] papers: FOG development was associated with HAMD-A, but not HAMA scores. We considered this a positive relationship between anxiety and FOG for the purposes of this literature review, but these discordant findings between scales that are designed to measure similar symptoms speak to the issue of variable anxiety detection as a function of scale. In addition, these contrast with Ou et al. [21], Lieberman [23], and Kostic et al.'s [31] results wherein HAMA scores were significantly higher in freezers than those in nonfreezers. It is thus still unclear whether FOG is significantly linked to HAMA scores.

Of the remaining scales, the STAI used in Ehgoetz Martens et al.'s study [32] has evidence of discriminant and convergent validity [51], but consensus exists in the literature that its applicability to PD needs to be investigated further [46, 47]. Interestingly, no significant differences between PD patients with and without FOG were illustrated by the baseline STAT-I in their study, but self-reported anxiety (collected on the manikin during the virtual reality task) was significantly higher in freezers than that in nonfreezers. This finding suggests that capturing anxiety in FOG is multifaceted and may require a more comprehensive anxiety assessment to adequately assess the phenomena. Similarly, Gilat et al. [42] acknowledge in their paper that their finding of no significant group difference between freezers and nonfreezers in Item 21 of PDQ-39 may be secondary to not thoroughly assessing the baseline mood disturbance of the participants. Using this single 5-point question to quantify anxiety is limited in scope, and more comprehensive measures should be used when assessing anxiety in this context.

Finally, Leentjens et al. [49] developed the Parkinson Anxiety Scale (PAS) used by Ehgoetz Martens et al. [29]. This is the most recently developed anxiety scale of the six and reportedly possesses superior validity and encompasses PD-specific anxiety symptoms more effectively by assessing three types of anxiety: persistent, episodic, and avoidant. The significant association between PAS scores and the anxious subgroup of gait freezers identified by Ehgoetz Martens et al. [29] warrants further study to further explore the validity and reliability of this scale. The Geriatric Anxiety Inventory is another scale that has been recommended specifically for use in PD patients [47] but was not used by the studies in this literature review. In light of neuroimaging data that suggest the HAMA and STAT-I measure different aspects of anxiety [52] (with the HAMA related to the subclinical expression of anxiety disorders and the STAT-I related to underlying personality characteristics conducive to anxiety), it is important to implement a scale that fully accounts for different anxiety variants, standardize the anxiety outcome measure that is ultimately used, and ensure its construct validity is suitable for PD patients with FOG in future research.

### 4.3. FOG

Thirteen studies implemented differing approaches to FOG identification, in that FOG was deemed present by either self-reported measures, family member accounts, or direct neurologist observation. This variable approach to detection stems from the episodic nature of FOG, a characteristic which reportedly makes detection of the phenomena difficult, impedes standardization of assessment, and limits generalizability of research [53]. In keeping with this, multiple studies in this review list recall bias secondary to self-reported measures of FOG as a limiting factor in their respective papers [18, 20, 21]. Although portions of the UPDRS and FOG-Q are convenient, valid, and widely used tools for assessing FOG symptoms in large numbers of participants, the potential for observation bias is well documented [46]. Seven papers in this review implemented the nFOG-Q, a scale largely based on the FOG-Q which shares the same susceptibility to recall bias. However, Nieuwboer et al. [17] report that the nFOG-Q is a reliable tool to screen FOG and can detect FOG that has previously occurred in the home environment—a benefit not conferred by experimental paradigms which elicit FOG in a laboratory setting.

Six of the studies [25, 28, 29, 32, 36, 42] approached the issue of FOG detection by combining self-reported measure with direct observation in a laboratory setting. This approach is in keeping with recommendations of Nonnekes et al. [54], who posit that both objective and subjective measures of FOG are necessary to account for the multifactorial nature by which FOG presents. This also allows for an appropriate degree of standardization. Each study implemented a different protocol for their objective FOG measurements, and all six corroborated their experimental findings with self-reported measures. In this subset of studies, four out of six demonstrated a significant relationship between FOG and anxiety. These studies are especially innovative with their various approaches to objectively measuring and at times consistently inducing FOG.

A standardized method of FOG elicitation and assessment that combined subjective and objective measures would allow more direct comparisons between studies to be drawn. Notably, the newly designed C-FOG used by Ehgoetz Martens et al. [29] delineates multiple phenotypes of FOG, and the significant findings of this paper warrant attention. A more comprehensive and nuanced assessment of FOG presentation, standardization of these scales in future research on anxiety and FOG, and consistency in outcome measurements mark important future directions of research that could identify anxiety as a potential treatable biomarker in the pathogenesis of FOG [29].

### 4.4. Neural Correlates

Neuroimaging research included in the review largely supports the implication of anxiety as both a prodromal symptom of PD and an important independent contributor to FOG pathogenesis. The cross-talk model suggests that increased limbic load (secondary to higher levels of anxiety and competing inputs) could precipitate FOG by overloading the striatum, interfering with normal basal ganglia motor processing [55]. This model aligns with the neuroimaging results from the study of Ehgoetz Martens et al. [28], Gilat et al. [42], and Rubino et al. [38]. Ehgoetz Martens et al. [28] characterized three unique phenotypes of FOG using behavioural measures and neuroimaging of the limbic corticostriatal pathway [28], Gilat et al. [42] demonstrated enhanced baseline striato-limbic connectivity in freezers vs. nonfreezers [42], and Rubino et al. [38] found evidence for volume loss in the posterior parietal cortex contributing to FOG. Most pertinent to this literature review, HADS scores in the study of Ehgoetz Martens et al. [28] were significantly associated with increased coupling between the limbic network and ventral striatum/cognitive control network within the anxious subtype of FOG. Future investigations should further ascertain the degree to which anxiety impacts functional connectivity in FOG.

## 5. Conclusion

A growing body of literature has linked anxiety to the presence, severity, and progression of FOG in PD patients, adding a neuropsychiatric element to what was once thought to be a solely motor phenomenon. The majority of the observational studies in this literature review with anxiety as a primary outcome relate anxiety to FOG, and these results are supplemented by experimental paradigms where anxiety-inducing environments reliably elicit FOG, anxiety levels significantly predict response to pharmacological management of FOG, and anxiety levels predict specific FOG phenotypes based on neuroimaging. A review of this literature reveals conflicting findings perhaps attributable to variable and potentially suboptimal anxiety and FOG measures in PD. Establishing a standardized anxiety scale suitable for PD should be of primary importance in future research on this topic. Recent successes with evoking FOG in an experimental setting in tandem with self-reported measures are encouraging. Future studies should incorporate both self-reported and objective assessments of FOG. The latter should be investigated in settings that have potential to induce FOG, increasing the frequency with which this symptom can be observed. Furthermore, the current state of evidence does not permit causal statements regarding the relation between anxiety and FOG. Future designs should aim to expose and critically test the nature of this relation between these two variables. Understanding the determinants and underpinnings of FOG is important as this could lead to novel treatment options for this relatively intractable symptom that is minimally responsive to dopaminergic or surgical therapies.

## Figures and Tables

**Table 1:** Inclusion and exclusion criteria.

Inclusion criteria	Exclusion criteria
(i) English language (ii) Peer-reviewed (iii) Participants with confirmed diagnosis of PD (iv) Compared both anxiety and FOG as primary outcome measures (v) Articles published up to September 19, 2018	(i) No exploration of anxiety and/or FOG as outcome measures (ii) Assessed festination or other gait disorders and did not document measures assessing FOG (iii) Review articles (iv) Case reports (v) Conference abstracts and nonpublished articles

**Table 2:** Summary of studies included in literature review.

Authors	Purpose and design	Demographic characteristics	PD measures	Anxiety measures	FOG measures	Notable results
Zhang et al. [18]	(i) A 3-year prospective longitudinal study where suspected risk factors for FOG in patients with early-stage PD are tabulated (ii) Incidence of FOG assessed at one, two, and three years following study initiation to evaluate suspected risk factors (including anxiety) associated with future development of FOG (iii) Participants recruited from the Third People's Hospital of Huzhou, Zhejiang Province of China	(i) Chinese population (ii) 248 PD patients (a) 112 males; 136 females (b) <65 years old (*n* = 78) mean age: 58.22 ± 5.43 (c) >65 years old (*n* = 170) mean age: 69.42 ± 3.41 (iii) Inclusion criteria: early-stage PD patients, never experienced FOG prior to study initiation, and never taken anti-Parkinson drugs (iv) Exclusion criteria: presence of atypical or secondary parkinsonism	(i) All participants diagnosed in accordance with the UK Brain Bank clinical criteria (ii) UPDRS-III and H&Y stage used to assess severity of PD motor symptoms (iii) UPDRS-III score (a) All 248 patients met the criteria (b) Mean: 19.22 ± 2.61 (iv) H&Y stage: (a) Stage 1 (*n* = 56) (b) Stage 1.5 (*n* = 135) (c) Stage 2 (*n* = 55) (d) Stage 2.5 (*n* = 2)	(i) Patients diagnosed by at least one trained psychiatrist and who had completed both the HAMA and HAMD Anxiety subscale (ii) At baseline, 51 PD patients were diagnosed with anxiety, and 3 years later, (a) 27 of these individuals developed FOG (b) 24 of these individuals did not develop FOG (iii) PD patients with FOG 3 years later (*n* = 128) (a) Overall HAMA: 11.35 ± 7.12 (b) HAMA-Somatic Anxiety: 4.81 ± 3.28 (c) HAMA-Psychic anxiety: 6.54 ± 3.96 (d) HAMD-Anxiety/Somatic: 2.79 ± 1.75 (iv) PD patients without FOG 3 years later (*n* = 120) (a) Overall HAMA: 10.23 ± 7.77 (b) HAMA-Somatic Anxiety: 4.44 ± 3.50 (c) HAMA-Psychic Anxiety: 5.79 ± 3.49 (d) HAMD-Anxiety/Somatic: 2.26 ± 1.43 (v) The paper mentions some patients were on anxiolytics but does not report numbers	(i) A convincing subjective report of FOG in addition to patient's recognition of FOG phenomenon when demonstrated by an experienced clinician. OR (ii) Answered yes to FOG-Q3OR (iii) Reported by patients, their family members, or caregivers when occurring at home. (a) FOG after 1 year = 40 (b) FOG after 2 years = 98 (c) FOG after 3 years = 128	Following age-adjusted ANCOVA: (i) No significant association with HAMA scores at baseline and FOG 3 years later (ii) Significantly higher HAMD anxiety scores in FOG compared with non-FOG patients 3 years later (*p* = 0.002) Following binary logistic regression analysis: (i) Baseline HAMD Anxiety significantly associated with future development of FOG 2 years later (*p*=0.033) but not 1 year later (*p*=0.217)

Martens et al. [19]	(i) To compare anxiety levels of PD patients with FOG directly with PD patients without FOG, as well as how anxiety contributes to FOG (ii) Participants recruited from a larger cohort of cases prospectively evaluated between 2008 and 2015 and divided into groups based on presence and severity of FOG and various clinical variables tabulated and compared including anxiety	(i) Australian population (ii) 461 PD patients at the University of Sydney-affiliated PD Research Clinic recruited and split into 3 groups: (iii) PD without FOG (PDNF) (*n* = 231) (a) 150 males; 81 females (b) Mean age: 67.8 ± 0.64 (c) Mean PD duration: 3.8 ± 0.56 years (ii) PD with mild FOG (PDmFOG) (*n* = 50) (a) 34 males; 16 females (b) Mean age: 69.3 ± 1.09 (c) Mean PD duration: 6.3 ± 0.87 years (iii) PD with FOG (PDFOG) (*n* = 180) (a) 109 males; 71 females (b) Mean age: 69.2 ± 0.77 (c) Mean PD duration: 8.7 ± 0.6 years (iv) Exclusion criteria: presence of neurological disease other than PD, psychiatric disorders other than affective disorder, and MMSE <24	(i) All participants had confirmed diagnosis of idiopathic PD by a trained neurologist based on UK Brain Bank clinical criteria (ii) PDNF (a) Mean UPDRS-III: 28.3 ± 1.1 (iii) PDmFOG (a) Mean UPDRS-III: 33.5 ± 2.5 (iv) PDFOG (a) Mean UPDRS-III: 41.55 ± 1.3 (v) Significant group differences in above scores observed in ANOVA (*p* < 0.001), further covariate analysis performed to factor UPDRS-III score out of outcome measures analysis (in addition to MMSE score and age)	(i) Anxiety subscale of HADS (HADS-A) subtotalled and compared between groups with scores ≥ 8 meeting criteria for anxiety disorder (ii) PDNF (a) Mean HADS-A: 3.5 ± 0.22 (iii) PDmFOG (a) Mean HADS-A: 4.7 ± 0.55 (iv) PDFOG (a) Mean HADS-A: 5.8 ± 0.31 (v) To determine whether or not HADS-A could predict FOG, PDmFOG group combined with the PDFOG group and HADS-A and HADS-D scores between the two groups compared: (a) PDNF (231 total): 14 patients with anxiety and 10 patients with anxiety and depression (b) PDFOG combined (230 total): 26 patients with anxiety and 39 patients with anxiety and depression (vi) 129 patients in this study taking anxiolytics and/or antidepressants (a) 62/231 PDNF (b) 13/50 PDmFOG (c) 54/180 PDFOG	(i) FOG-Q3 score used to differentiate participants into 3 groups: (ii) PDNF (a) FOG-Q3: 0 (iii) PDmFOG (a) FOG-Q3: 1 (iv) PDFOG (a) FOG-Q3: 2–4	(i) An ANCOVA conducted across groups demonstrated that, compared to PDNF, PDFOG patients had significantly greater anxiety (*p* < 0.001); PDmFOG was not significantly different from PDFOG (ii) Pearson's correlations with stepwise multiple regression analyses demonstrated that, in all groups, severity of anxiety symptoms was significantly correlated with FOG-Q3 scores (*p* < 0.001) (iii) In PDFOG combined compared with PDNF, a significant odds ratio of 3.40 (*p* < 0.001) when anxiety was exposure outcome and FOG was the variable (ii) HADS-A score PPV of 28.26% (95% CI 22.26%–34.35%) for identifying patients with FOG (iii) HADS-A score NPV of 89.61% (95% CI: 84.94%–93.23%) for identifying patients without FOG

Walton et al. [20]	(i) To examine the impact of FOG on the Health-Related Quality of Life scale in PD patients (ii) Additionally, other factors that are known to impact patient well-being were assessed (including anxiety) (iii) Data collected from baseline assessment of a group of PD patients from December 2008 and November 2013 patients at the University of Sydney-affiliated PD Research Clinic	(i) Australian population (ii) 203 PD patients (a) 139 males; 64 females (b) Mean age: 66.77 ± 8.9 (c) Mean PD duration: 61.30 ± 61.3 months (iii) Excluded if MMSE <24	(i) All participants had confirmed diagnosis of idiopathic PD based on United Kingdom Brain Bank clinical criteria by a trained neurologist (ii) Mean UPDRS-III score: 27.60 ± 15.1 (iii) H&Y stage (a) Stage 1: 39 (19.21%) (b) Stage 1.5: 7 (3.45%) (c) Stage 2: 98 (48.28%) (d) Stage 2.5: 39 (19.21%) (e) Stage 3: 20 (9.85%)	HADS-A score quantified: (a) Mean: 3.95 ± 3.4	(i) FOG-Q3 score used to identify patients that had experienced FOG: (a) 86 patients ≥1 (43% FOG) (ii) Full 6-Item FOG-Q used to assess severity of FOG: (b) Mean score: 5.34 ± 5.7	(i) Pearson's bivariate correlation indicated HADS-A score significantly associated with severity of FOG (*p* < 0.01)

Ehgoetz Martens et al. [32]	(i) To induce anxiety via virtual reality and measure presence/severity of FOG in a controlled experimental setting (ii) 10 randomized walking trials across a 6-metre long plank positioned between two platforms in one of the two different virtual 3-D environments: a low condition wherein the plank was positioned on the floor of the virtual environment, and a high condition wherein the plank was positioned over a pit 8 metres in depth of the virtual environment (iii) Patients recruited through a Wilfrid Laurier University affiliated Research and Rehabilitation Centre in Waterloo, Canada	(i) Canadian population (ii) 31 PD patients divided into freezers and nonfreezers: (iii) 14 freezers: (a) 11 males; 3 females (b) Mean age: 71 ± 7.8 (iv) 17 nonfreezers: (a) 14 males; 3 females (b) Mean age: 66 ± 8.7 Exclusion criteria: unable to walk 10 m unassisted, severe vertigo, motion sickness, severe kyphosis, other neurological disorders, severe head tremor, or dyskinesias	(i) Previous diagnosis of idiopathic PD by a neurologist (ii) Mean UPDRS-III score: (a) Freezers: 34 ± 10.1 (b) Nonfreezers: 20 ± 10.4	(i) State and Trait Anxiety Inventory Scale assessed baseline levels of anxiety prior to completing experiment: (ii) Freezers (a) Average STAI-Trait: 33 ± 6.9 (b) Average STAI-State: 34 ± 8.7 (iii) Nonfreezers (a) Average STAI-Trait: 32 ± 6.6 (b) Average STAI-State: 30 ± 5.9 (iii) Participants additionally completed a 9-point self-assessment manikin scale after each walking trial to quantify anxiety experienced during the experiment	(i) Previous history of FOG and self-reported FOG using the UPDRS-II and FOG confirmed by movement disorder specialist prior to participation in study differentiated freezers from nonfreezers (ii) Freezers vs. nonfreezers (a) Low condition: % of each trial frozen = 11.03 ± 23 seconds (b) High condition: % of each trail frozen = 23.1 ± 28.7	(i) Freezers spent a significantly greater portion of time frozen in the high condition compared to the low condition (*F*(1,13) = 11.35, *p* < 0.005) (ii) Freezers exhibited significantly higher numbers of freezing episodes in the high condition relative to the low condition (*F*(1,13) = 8.29, *p* < 0.013), but no significant differences in duration of freezing episodes (*F*(1,13) = 2.48, *p*=0.14) (iii) Freezers self-reported significantly higher levels of anxiety during the experiment than nonfreezers (*F*(1,29) = 16.96, *p*=0.0003), with both groups reporting significantly higher levels of anxiety in the high condition compared to the low condition (*F*(1,29) = 29.83, *p* < 0.001) (iv) No significant differences between freezers and nonfreezers in terms of STAI-Trait (*p*=0.37) and STAI-state scores (*p*=0.20)

Ou et al. [21]	(i) To explore the prevalence and clinical correlates of FOG (ii) A cross-sectional observational study and participants were recruited from Department of Neurology, West China Hospital of Sichuan University between May 2011 and April 2014	(i) Chinese population (ii) 474 PD patients (iii) 221 with FOG (a) 117 males; 104 females (b) 108 ≤ 65 years old (c) 113 ≥ 65 years old (d) Mean age: 64.36 ± 10.16 (e) Mean PD duration: 5.97 ± 4.24 years (iv) 252 without FOG (a) 140 males; 113 females (b) 155 ≤ 65 years old (c) 98 ≥ 65 years old (d) Mean age: 60.11 ± 10.50 (e) Mean PD duration: 3.73 ± 3.55 years (v) Atypical or secondary parkinsonism excluded	(i) Patients previously diagnosed with UK Brain Bank criteria for PD (ii) H&Y stage and UPDRS-III used to assess severity (iii) Freezer H&Y stage (a) Stage 1–2.5: 115 (b) Stage 3: 74 (c) Stage 4-5: 32 (d) Median: 2.5 (1.0) (iv) Nonfreezer H&Y stage (a) Stage 1–2.5: 228 (b) Stage 3: 20 (c) Stage 4-5: 3 (d) Median: 2.0 (0.5) (v) Mean UPDRS-III score: (a) Freezers: 35.90 ± 14.48 (b) Nonfreezers: 24.10 ± 10.45	(i) HAMA scores quantified for freezers vs. nonfreezers: (a) Mean HAMA freezers: 9.64 ± 7.50 (b) Mean HAMA nonfreezers: 6.21 ± 5.92	(i) Freezing episodes observed by the neurologist during visit or reported by patient/family/caregiver if happened at home (ii) FOG-Q3 is used to assess	(i) 46.62 % prevalence of FOG in this population of PD patients (ii) ANCOVA adjusted for age, disease duration, and H&Y stage total HAMA scores significantly higher in freezers than nonfreezers (*p* < 0.001) (iii) Binary logistic regression model indicated no significant correlation between HAMA anxiety score and FOG (*p*=0.333)

Perez-Loret et al. [34]	(i) To determine prevalence of FOG and assess its relationship with quality of life and other clinical factors (including anxiety) (ii) Cross-sectional study (iii) Patients included prospectively as outpatients of public or private practicing neurologists in 32 centres from 4 different regions of France	(i) French population (ii) 672 patients: (a) 381 males; 291 females (b) Age ≥ 68: 341 (c) PD duration ≥ 5 years: 341 (iii) Exclusion criteria: patient's <18 years old, MMSE <24, undergoing deep brain stimulation, and having serious disease affecting life expectancy in the short term	(i) Each PD patient examined by the neurologist with standardized/structured interview	(i) Anxiety subscale of HADS (HADS-A) subtotalled, with a number of PD patients with scores ≥ 7 totalled and compared between freezers and nonfreezers (ii) Freezers (a) Number of PD patients with HADS-A ≥ 7: 136 (iii) Nonfreezers (a) Number of PD patients with HADS-A ≥7: 194	(i) FOG identified by scores ≥1 on Item 14 of UPDRS-II (ii) Nonfreezers: 415 (iii) Freezers: 257	(i) 38.2% FOG point prevalence reflecting a mixed population of patients with early and advanced disease who were still ambulatory, with or without motor fluctuations (ii) No significant differences between HADS-A anxiety in freezers vs nonfreezers

Burn et al. [22]	(i) To identify associations between phenotypic variants of motor and mood subtypes of PD (ii) Cross-sectional study (iii) Participants were recruited over a period of 12 months from specialist PD or movement disorder outpatient clinics as part of the PROMS-PD longitudinal study	(i) UK population (ii) 513 PD patients (iii) Phenomenological data relating to depression and anxiety collected by trained staff using the Geriatric Mental State semi-structured interview and sorted into 4 groups: (a) Anxious-depressed (8.6%) (b) Depressed (9.0%) (c) Anxious (22%) (d) Low-level psychiatric symptomatology (60.4%) Inclusion criteria: able to provide informed consent and resided within 2 hours travel time of a study research centre Exclusion criteria: the presence of communication difficulty or sensory deficits that interfered with assessment. Cognitive impairment and severe psychiatric disorders only met exclusion criteria if they were severe enough to affect capacity to consent	(i) Patients with a previous diagnosis of PD recruited from specialist movement disorder and care of the elderly clinics (ii) Freezer H&Y stage (a) Stage 1–2.5: 115 (b) Stage 3: 74 (c) Stage 4-5: 32 (d) Median: 2.5 (1.0) (iii) Nonfreezer H&Y stage (a) Stage 1–2.5: 228 (b) Stage 3: 20 (c) Stage 4-5: 3 (d) Median: 2.0 (0.5) (iv) Mean UPDRS-III score: (a) Freezers: 35.90 ± 14.48 (b) Nonfreezers: 24.10 ± 10.4	(i) Anxiety subscale of HADS (HADS-A) subtotalled with scores ≥ 11 meeting criteria for significant anxiety symptoms (22.0%) (ii) Number of PD patients with HADS ≥ 11 (a) Freezers: 25 (33%) (b) Nonfreezers: 87 (20%) (iii) Mean total HADS-A Score (a) Freezers: 8.8 ± 4.6 (b) Nonfreezers: 6.9 ± 4.4	(i) FOG identified by scores >1 on Item 14 of UPDRS-II (a) FOG present: 75 patients (14.62%) (b) FOG not present: 438 patients (85.38%)	(i) The mean total HADS-A scores in freezers was significantly higher than in nonfreezers (*p* < 0.001), with a significantly higher proportion of freezers meeting the HADS-A ≥ 11 criteria for anxiety than nonfreezers (*p* < 0.017) (ii) Presence of FOG did not predict membership in the anxious-depressed, depressed, or anxious mood subtypes

Lieberman [23]	(i) To study the association between anxiety, depression, and panic with FOG in Parkinson's patients	(i) US population (ii) 109 patients: (a) 72 males; 37 females (b) Mean age: 69.0 ± 11.4 (c) PD duration: 7.6 ± 5.7 (iii) Patient's completed the Activities of Daily Living section of the UPDRS which contains a question about freezing	(i) Patients previously diagnosed with PD	(i) HAMA scores quantified with score ranges of 18–54 meeting criteria for anxiety (a) 18/29 with FOG (62%) (b) 14/80 without FOG (18%) (ii) DSM-IV criteria for panic attacks used to identify presence of panic attacks (a) 14/29 with FOG (48%) (b) 6/80 without FOG (80%)	(i) Those that answered yes to the initial freezing question (item 14 of UPDRS-II) further questioned and evaluated using 6 Item FOG-Q to calculate FOG severity	(i) FOG was significantly associated with anxiety (*p* < 0.002) and panic attacks (*p* < 0.0001)

Hall et al. [35]	(i) Evaluate domains of motor, cognitive, affective, autonomic, and REM sleep behaviour disorder in early-stage PD and their respective associations with FOG (ii) Data collected from baseline assessment of a group of PD patients from December 2008 and November 2013 at the University of Sydney-affiliated PD Research Clinic	(i) Australian population (ii) 91 PD patients (iii) Freezers (*n* = 38) (a) 27 males; 11 females (b) Mean age: 65.20 ± 9.00 (c) Mean PD duration: 1.91 ± 1.40 years (iv) Nonfreezers (*n* = 53) (a) 35 males; 18 females (b) Mean age: 64.58 ± 8.63 (c) Mean PD duration: 1.69 ± 1.26 years (v) Included if a H&Y stage <3 and a disease duration of 5 years or less (vi) Excluded on the presence of other neurological diseases, conditions that would impair gait, or presence of dementia as rated by Movement Disorder Task Force criteria (vii) No significant differences in age, years of education, and disease duration between freezers and nonfreezers	(i) All participants had confirmed diagnosis of idiopathic PD based on United Kingdom Brain Bank clinical criteria by a trained neurologist (ii) Freezers (a) Mean H&Y stage: 2.00 ± 0.42 (b) Mean UPDRS-III: 25.66 ± 14.11 (iii) Nonfreezers (a) Mean H&Y stage: 1.85 ± 0.44 (b) Mean UPDRS-III: 20.85 ± 10.70	(i) HADS-A scores quantified for freezers vs. nonfreezers: (a) Mean HADS-A freezers: 4.92 ± 4.58 (b) Mean HADS-A nonfreezers: 3.42 ± 2.49	(i) FOG-Q3 used to separate freezers from nonfreezers (a) ≥1: 38 freezers (41.76%) (b) 0: 53 nonfreezers (58.24%)	(i) Despite higher mean scores on the HADS-A scale, no significant differences were noted between anxiety scores of freezers compared with nonfreezers (*p*=0.800)

Yao et al. [24]	(i) Exploring whether cognitive impairment is correlated with occurrence of FOG in PD neuropsychological assessments utilized to evaluate cognitive functioning: SCOPA-COG, UPDRS, MMSE, HAMA, and HAMD. (ii) Data collected from PD patients diagnosed and treated in YangPu, Nanjing Drum Tower and Huashan hospitals (iii) Case-control study	(i) Chinese population (ii) 186 patients with PD (iii) FOG PD patients (*n* = 104) (a) 55 males; 49 females (b) Mean age: 65.72 ± 7.34 (c) Mean PD duration: 9.52 ± 1.54 years (d) Years of education: 13.51 ± 4.24 (iv) Non-FOG PD patients (*n* = 82) (a) 44 males; 32 females (b) Mean age: 66.35 ± 8.67 (c) Mean PD duration: 9.16 ± 1.27 years (d) Years of education: 12.82 ± 3.67 (v) 125 health controls (a) 66 males; 59 females (b) Mean age: 65.91 ± 6.86 (c) Years of education: 13.32 ± 2.93 (vi) Inclusion criteria: disease duration <10 years, MMSE ≥24, H&Y stage ≤2.5, signs of stabilization with antiparkinsonism treatment prior to study (vii) Exclusion criteria: significant comorbidities exhibit signs of depression/anxiety, patients diagnosed with cardiovascular and/or cerebrovascular disease, anticholinergic treatments, orthopaedic or additional neurological disorders (PD and control group subjects matched for gender, age, year of education, and course of disease)	(i) After diagnosis by the neurologist, a total of 186 PD patients were selected for the study (ii) FOG PD patients (*n* = 104) (a) UPDRS-I: 4.53 ± 1.24 (b) UPDRS-II: 10.64 ± 3.42 (c) UPDRS-III: 13.71 ± 4.16 (d) H&Y rating: 2.01 ± 0.26 (e) Levodopa therapy, *n*: 62 (59.62%) (f) Dopamine agonist therapy, *n*: 42 (40.38%) (iii) Non-FOG PD patients (*n* = 82) (a) UPDRS-I: 3.32 ± 1.54 (b) UPDRS-II: 6.85 ± 3.32 (c) UPDRS-III: 9.27 ± 3.81 (d) H&Y rating: 1.92 ± 0.31 (e) Levodopa therapy, *n*: 51 (62.2%) (f) Dopamine agonist therapy, *n*: 31 (37.8%)	(i) Mean HAMA scores (including somatic/psychic anxiety subdomains) and the HAMD-anxiety/somatic subdomain were quantified and compared between FOG and non-FOG PD patients (ii) FOG PD patients: (a) Overall HAMA: 11.14 ± 3.71 (b) HAMA-Somatic Anxiety: 5.20 ± 2.51 (c) HAMA-Psychic Anxiety: 7.14 ± 3.12 (d) HAMD-Anxiety/Somatic: 3.12 ± 1.12 (iii) Non-FOG PD patients: (a) HAMA: 9.96 ± 3.21 (b) HAMA-Somatic Anxiety: 4.93 ± 2.60 (c) HAMA-Psychic Anxiety: 6.23 ± 3.43 (d) HAMD-anxiety/somatic: 2.67 ± 1.03 (iv) HAMD-Anxiety scale score compared with severity of FOG within FOG patients (v) The paper does not document incidence of anxiolytic treatment	(i) FOG identified by 2/3 criteria used by Snijders et al. (2011): (a) Convincing subjective reports of FOG (b) Patient's subjective recognition of FOG when demonstrated by an experienced clinician (c) Scores ≥1 on nFOG-Q (ii) nFOG-Q used to assess severity of FOG	(i) Two-tailed *t*-test comparing HAMA scores between FOG and non-FOG PD patients showed higher mean scores for the FOG group but no statistically significant differences between groups. (ii) Higher mean scores and significant differences between the HAMD-Anxiety/Somatic scale were observed between groups: overall HAMA (*p*=0.016), HAMA-Somatic Anxiety (*p*=0.47), HAMA-Psychic Anxiety (*p*=0.06), and HAMD-Anxiety (*p*=0.005) (iii) Spearman's rho correlation between HAMD-Anxiety and FOG questionnaire demonstrated severity of FOG positively correlated with anxiety within the FOG group: (a) *r* = 0.79 (*p* < 0.0001)

Rahimi et al. [25]	(i) A pilot study wherein optimally medicated PD patients with intractable FOG were treated with a 90-day course of 1 mg rasagiline, with FOG and cognitive/motor measures assessed prior to and following treatment (ii) UPDRS motor section (Items 18–31), MoCA, BAI, BDI, FOG-Q, 6 scripted gait tasks, and free walking task administered pre- and posttreatment. Gait tasks were video-recorded with subsequent analysis by two independent examiners (iii) Prospective, experimental, pre-/posttreatment design, uncontrolled design (iv) Participants recruited from a movement disorders clinic in London, on using the convenience-sampling method	(i) Canadian population (ii) 14 participants completed entire study protocol (out of 18 total); PD patients separated into 3 groups upon completion of rasagiline therapy and subsequent analysis: improved, worsened, and no change (iii) Improved (*n* = 6) (a) 5 males; 1 females (b) Mean age: 69.7 ± 3.88 (c) Mean PD duration: 9.8 ± 3.13 years (d) Mean FOG duration: 5.2 ± 2.64 years (iv) Worsened (*n* = 5) (a) 4 males; 1 females (b) Mean age: 67.8 ± 7.40 (c) Mean PD duration: 12.0 ± 5.34 years (d) Mean FOG duration: 4.0 ± 2.00 years (ii) No change (*n* = 3) (a) 3 males, 0 females (b) Mean age: 67.8 ± 7.40 (c) Mean PD duration: 15.67 ± 7.02 years (d) Mean FOG duration: 5.0 ± 2.00 years (v) No significant differences among groups for any of the demographic or baseline variables between the 3 groups (*p* values ≥0.10) (vi) Inclusion criteria: H&Y scores between 2 and 4, experienced intractable FOG even with optimal dopaminergic treatment, on stable clinically optimated medications for 3 months prior and were clinically determined to require adjunct therapy with rasagiline (vii) Exclusion criteria: history of neurological procedures, other neurological disorders, untreated clinical depression, diagnosed GAD, dementia, and use of a gait aid	(i) A movement disorders neurologist enrolled all participants during routine clinic visits. All enrolled patients met UK Brain Bank criteria for PD.	(i) BAI scale used to assess anxiety (ii) Improved (a) Mean pretreatment BAI score: 13.0 ± 7.43 (b) Mean posttreatment BAI score: 15.7 ± 8.69, (ii) Worsened (*n* = 5) (a) Mean pretreatment BAI score: 18.2 ± 9.15 (b) Mean posttreatment BAI score: 16.2 ± 10.59 (iii) No change (*n* = 3) (a) Mean pretreatment BAI score: 12.7 ± 9.24 (b) Mean posttreatment BAI score: 13.7 ± 15.18	(i) FOG diagnosed by movement disorders neurologist through gait analysis in clinic, patient report corroborated by a family member, and clinical history, UPDRS scores, and FOG-Q scores administered at the first visit	(i) No significant differences in pre-/posttreatment BAI scores (*t*(13) = 0.26, *p*=0.80), or overall count and duration of FOG episodes (*F*(1,13) = 0.96, *p*=0.34; *F*(1,13) = 1.00, *p*=0.34, respectively) (ii) Higher BAI anxiety scores predicted FOG worsening following rasagiline therapy (*p* < 0.0024) (iii) Final predictive model explained 99% of the variance between the improved and worsened groups as a function of treatment with rasagiline

Huh et al. [36]	(i) Case control cross-sectional design (ii) Examined postural sensory deficits linked to FOG severity through a computerized dynamic posturography system (iii) Participants evaluated with a sensory organization test and several clinical measures (including anxiety) (iv) Participants recruited through Samsung Medical Centre (v) Clinical, neurological exams, and posturography were performed during the morning off-medication, at least 12 hours after holding Parkinson's medications	(i) South Korean population (ii) 47 patients with PD (iii) FOG PD patients (*n* = 25) (a) 13 males; 12 females (b) Median age: 64.0 (58.5–69.5) (c) Median PD duration: 10.0 (8.0–12.0) (d) Median years of education: 12.0 (12.0–16.0) (iv) Non-FOG PD patients (*n* = 22) (a) 13 males; 9 females (b) Median age: 64.5 (61.0–67.8) (c) Median PD duration: 9.0 (7.8–10.3) (d) Median years of education: 12.0 (9.0–16.0) (v) 26 healthy controls (a) 13M, 13F (b) Median age: 64.0 (62.0–66.0) (c) Median years of education: 12.0 (12.0–16.0) (vi) No significant differences between gender, age, and years of education between the 3 groups (vii) No significant different between years of PD duration between FOG PD patients and non-FOG PD patients Exclusion criteria: unable to stand safely unaided, history of falls in the last 12 months, dementia, visual disturbances, clinical signs of disorders affecting proprioception, musculoskeletal problems, orthostatic hypotension, neurosurgical interventions, history, or clinical signs of vestibular disease (data are shown as median (25^th^–75^th^ quartile))	(i) A diagnosis of idiopathic PD made by a specialist experienced in movement disorders based on UK Brain Bank Criteria. (ii) Median UPDRS-III score: (a) Freezers: 35.0 (33.0–38.3) (b) Nonfreezers: 36.0 (31.5–37.3) (c) Controls: 0 (0–2.0) (iii) Median H&Y stage: (a) Freezers: 2.5 (2.5–3.0) (b) Nonfreezers: 2.5 (2.0–3.0) (data are shown as median (25^th^–75^th^ quartile)	(i) BAI used to assess anxiety (ii) FOG PD patients: (a) Median: 7.0 (3.0–14.0) (iii) Non-FOG PD patients: (a) Median: 5.0 (3.0–12.3) (iv) Controls: (a) Median: 1.0 (0–2.0) (data are shown as median (25^th^–75^th^ quartile)	(i) nFOG-Q used to assign participants to the FOG group based on item 1 of the questionnaire (if they had experienced FOG during the past month), and if ≥ 1, FOG episodes were elicited and detected by 2 movement specialists using a 2-minute tight and rapid turning-in-place protocol (ii) FOG PD patients: (a) Median nFOG-Q: 12.0 (10.5–18.5) (iii) Non-FOG PD patients: (a) Median: 0 (data are shown as median (25^th^–75^th^ quartile)	(i) FOG patients and non-FOG patients had significantly higher BAI scores than the control group (*p* < 0.001) with no significant difference between groups (*p*=0.980)

Ehgoetz Martens et al. [28]	(i) Prospective longitudinal study design (ii) PD patients were separated into 3 groups based on their responses to FOG-Q3 at baseline and a follow-up point 6–24 months later, with measures of anxiety and FOG severity taken at both of these time points (iii) Participants recruited from University of Sydney's PD Research Clinic at the Brain and Mind Centre from 2008 to 2016	(i) Australian population (ii) 221 PD patients (iii) Continuing freezer (*n* = 92) (a) 56 males; 36 females (b) Mean age: 70.24 ± 10.85 (c) Mean PD duration: 9.66 ± 7.50 years (iv) Transitional freezer (*n* = 41) (a) 26 males; 15 females (b) Mean age: 70.75 ± 9.39 (c) Mean PD duration: 5.97 ± 4.14 years (v) Nonfreezers (*n* = 88) (a) 52 males; 36 females (b) Mean age: 65.37 ± 9.84 (c) Mean PD duration: 3.16 ± 4.32 years (vi) Significant group effects for age (*p*=0.002) and disease duration (*p* < 0.001) found and therefore entered as covariates in subsequent analysis	(i) A diagnosis of idiopathic PD made by a specialist experienced in movement disorders based on UK Brain Bank Criteria. (ii) Mean baseline UPDRS-III scores: (a) Continuing freezers: 48.64 ± 20.68 (b) Transitional freezers: 35.14 ± 17.07 (c) Nonfreezers: 24.51 ± 13.62 (iii) Mean follow-up UPDRS-III scores: (a) Continuing freezers: 49.81 ± 21.06 (b) Transitional freezers: 38.42 ± 15.33 (c) Nonfreezers: 25.51 ± 14.40	(i) HADS-A used to assess anxiety (ii) Mean baseline HADS-A scores: (a) Continuing freezers: 2.94 ± 3.89 (b) Transitional freezers: 3.65 ± 3.08 (c) Nonfreezers: 1.18 ± 2.31 (iii) Mean follow-up HADS-A scores: (a) Continuing freezers: 5.38 ± 4.19 (b) Transitional freezers: 5.18 ± 4.68 (c) Nonfreezers: 3.62 ± 2.93	(i) FOG-Q3 used to assign participants to groups based on scores, FOG-Q total used to assess severity (with Item 3 removed) (ii) Mean FOG-Q total scores: (a) Continuing freezers: 10.23 ± 4.19 (b) Transitional freezers: 2.68 ± 1.74 (c) Nonfreezers: 1.41 ± 1.37 (iii) Mean follow-up FOG-Q total scores: (a) Continuing freezers: 10.79 ± 4.02 (b) Transitional freezers: 6.17 ± 2.39 (c) Nonfreezers: 1.68 ± 1.60	(i) HADS-A scores increased significantly from baseline to follow-up across all 3 groups, and both continuing and transitional freezers had significantly greater mean HADS-A scores than nonfreezers at baseline and follow-up (ii) Correlational analysis revealed HADS-A scores were significantly associated with FOG-Q total scores (0.206, *p* < 0.01)—predictive model constructed with HADS-A and FOG-Q total score as variables with the overall predictive success of 82.1%

Ou et al. [27]	(i) Prospective longitudinal study design (ii) PD patients were assessed at baseline and at follow-up 2.5–3 years later to investigate potential clinical predictors of FOG, which included anxiety (iii) Participants recruited from West China Hospital of Sichuan's Department of Neurology between March 2012 and May 2013	(i) Chinese population (ii) 225 PD patients (iii) 85 developed FOG after 3 years (a) 44 males; 41 females (b) Mean age: 62.5 ± 11.1 (c) Mean PD duration: 6.8 ± 3.3 years (iv) 140 did not develop FOG after 3 years (a) 85 males; 55 females (b) Mean age: 58.1 ± 12.6 (c) Mean PD duration: 4.5 ± 2.4 years (v) Inclusion criteria: between H&Y stages I-III, no prior FOG (vi) Exclusion criteria: atypical or secondary Parkinsonism, unstable disease, participants who declined follow-up (vii) Freezers significantly older (*p*=0.009) and had significantly longer PD duration (*p* < 0.001) than nonfreezers on average and further analysis done with ANCOVA adjusted for age and disease duration	(i) A diagnosis of idiopathic PD made by a trained neurologist based on UK Brain Bank Criteria (ii) Mean baseline UPDRS-III scores: (a) Freezers: 32.9 ± 12.8 (b) Nonfreezers: 21.4 ± 10.5 (iii) Mean follow-up UPDRS-III scores: (a) Freezers: 44.3 ± 14.8 (b) Nonfreezers: 29.8 ± 11.2 (iv) Average change in UPDRS-III score (a) Freezers: 3.7 ± 3.4 (b) Nonfreezers: 3.2 ± 3.0 (v) Mean baseline H&Y stages: (a) Freezers: 2.4 ± 0.6 (b) Nonfreezers: 1.5 ± 0.6 (vi) Mean follow-up H&Y stages: (a) Freezers: 2.9 ± 0.8 (b) Nonfreezers: 2.2 ± 0.5	(i) HAMA used to assess anxiety (ii) Mean baseline HAMA scores: (a) Freezers: 10.3 ± 6.8 (b) Nonfreezers: 7.2 ± 5.8 (iii) Mean follow-up HAMA scores: (a) Freezers: 11.0 ± 6.9 (b) Nonfreezers: 7.7 ± 6.0 (iv) Average change in HAMA score (a) Freezers: −0.2 ± 1.9 (b) Nonfreezers: −0.2 ± 1.7	(i) FOG-Q3 ≥ 1 used to separate freezers from nonfreezers (ii) Freezing episodes observed by the neurologist during visit or reported by patient/family/caregiver if happened at home	(i) Higher mean HAMA scores in freezers than nonfreezers at both baseline and follow-up (*p* < 0.001) (ii) Annual change in HAMA did not differ significantly between groups (*p*=0.780)

Ehgoetz Martens et al. [26] (fMRI study)	(i) An experimental task-based fMRI study (ii) PD patients with FOG completed virtual reality gait paradigm while in 3T MRI scanner (iii) Baseline assessment data, including anxiety outcome measures, performed on all participants (vi) Gait observed during the virtual reality task (foot tapping) was compared between freezing episodes and normal foot-tapping using fMRI (v) Participants recruited from University of Sydney's Brain and Mind Centre	(i) Australian population (ii) 41 PD patients with FOG, 5 freezing episodes were required in the MRI scanner for effective modelling of freezing events: 20 met this criteria (MRI + group), while 21 did not (MRI group) (iii) MRI+ (a) 17M, 3F (b) Mean age: 66.45 ± 5.34 (iii) MRI− (a) 15 males; 16 females (b) Mean age: 68.75 ± 7.26 (iv) Inclusion criteria: FOG-Q3 ≥ 1, clinically evident FOG confirmed by a neurologist, ability to complete virtual reality task in MRI scanner after a minimum of 12 hours following intake of dopaminergic medication (off state) (v) Exclusion criteria: neurological comorbidities and presence of pathological lesions/abnormalities on structural imaging (vi) Notably MRI+ and − groups were matched in age, symptom severity, FOG-Q3, and anxiety	(i) A diagnosis of idiopathic PD made by a trained neurologist based on UK Brain Bank Criteria (ii) MRI+ (a) Mean UPDRS-III: 34.15 ± 12.19 (b) MRI− (c) Mean UPDRS-III: 33.62 ± 15.84	(i) HADS-A used to assess anxiety (ii) MRI+ (a) Mean HADS-A: 6.3 ± 3.25 (iii) MRI− (a) Mean HADS-A: 5.29 ± 3.98	(i) FOG-Q3 ≥ 1 an inclusion criteria for the study used to identify PD patients with FOG in this study, which was additionally confirmed by a neurologist (ii) Objective measures of FOG collected through virtual gait paradigm (iii) MRI + (a) Mean step time variability: 24.99 ± 7.04 s (b) Modal foot step latency: 0.38 ± 0.17 s (c) Overall time spent frozen: 0.14 ± 0.08% (iv) MRI− (a) Mean step time variability: 21.27 ± 8.32 s (b) Modal foot step latency: 0.64 ± 0.29 s (c) Overall time spent frozen: 0.07 ± 0.14% (v) HADS-total one of three variables used to calculate the Freezing Component Index which was compared to the freezing network signature collected via fMRI	(i) Higher HADS-A scores associated with increased coupling between limbic network and ventral striatum + cognitive control network during gait freezing compared to normal foot tapping

Gilat et al. [42]	(i) A resting state MRI study investigating functional connectivity differences in PD patients with and without FOG (ii) Baseline assessment data, including anxiety outcome measures, performed on all participants recruited through Oregon Health and Science University's Parkinson's Centre of Oregon clinic	(i) American population (ii) Data from 40 PD participants included in study (iii) 19 freezers (a) Gender demographics not documented (b) Mean age: 68.4 ± 7.2 (c) Mean PD duration: 9.31 ± 6.6 years (iv) 21 Nonfreezers (a) Gender demographics not documented (b) Mean age: 66.9 ± 7.6 (c) Mean PD duration: 6.01 ± 4.6 years (v) Inclusion criteria: participants able to stop taking all dopaminergic medication for 12 hours prior to the experiment (vi) Exclusion criteria were as follows: unable to safely walk 20 feet without an aid, previous joint replacement, musculoskeletal or vestibular disorder, claustrophobia, severe tremor, and presence of metal implants (vii) No significant differences were found between freezers and nonfreezers in terms of age (*t* = 0.645, *p*=0.523), disease duration (*t* = 0.019, *p*=0.985), and UPDRS-III (*t* = 1.63, *p*=0.111)	(i) A diagnosis of idiopathic PD by both a neurologist and movement disorders specialist (ii) Freezers (a) Mean UPDRS-III: 42.7 ± 14 (b) Mean H&Y stage: 2 ± 2 − 4 (iii) Nonfreezers (a) Mean UPDRS-III: 36.1 ± 11 (b) Mean H&Y stage: 2 ± − 3	(i) Items 21 and 9 of PDQ-39 administered for anxiety outcome measure (ii) Freezers mean PDQ-39: (a) Item 21: 1 ± 0 − 2 (b) Item 9: 1 ± 0 − 2 (iii) Nonfreezers mean PDQ-39: (a) Item 21: 1 ± − 0 − 3 (b) Item 9: 0 ± 0 − 2	(i) FOG-Q3 ≥ 1 used to separate freezers from nonfreezers (ii) FOG was objectively quantified through a turning-in-place task with inertial sensors, allowing the FOG ratio to be calculated for each participant (iii) Mean FOG ratio (a) Freezers: 3.21 ± 2.9 (b) Nonfreezers: 1.04 ± 0.73	(i) No significant differences were observed between freezers and nonfreezers for Item 21 of the PDQ-39 (*Z* = −0.520, *p*=0.603) (ii) A trend towards significance was exhibited for freezers compared to nonfreezers in terms of higher PDQ Item 9 scores (*z* = −1.79, *p*=0.703), but this ultimately was not significant (iii) Freezers had significantly higher FOG-ratios than nonfreezers (*t*(38) = 3.12, *p* < 0.001), and worsening FOG severity was associated with greater degrees of anticoupling between the amygdala and the frontoparietal attention control network

Ehgoetz Martens et al. [28]	(i) An experimental study to characterize subtypes of freezing of gait with a newly generated FOG questionnaire: the C-FOG (ii) Participants subdivided into 3 categories based on C-FOG results: asymmetric-motor, anxious, and sensory-attention (iii) Baseline assessment data, including anxiety outcome measures, performed on all participants	(i) Australian population (ii) 41 participants (iii) Anxious subgroup (*n* = 15) (a) 11 males; 4 females (b) Mean age: 66.8 ± 8 (c) Mean PD duration: 12.3 ± 3.6 years (iv) Asymmetric-motor subgroup (*n* = 13) (a) 8 males; 5 females (b) Mean age: 68.2 ± 9.3 (c) Mean PD duration: 8.6 ± 4.8 years (v) Sensory attention subgroups (*n* = 13) (a) 10 males; 3 females (b) Mean age: 70.5 ± 7.8 (c) Mean PD duration: 8.7 ± 4.1 years (vi) No inclusion or exclusion criteria directly specified by paper (vii) Apart from significantly higher mean UPDRS-III scores in the off state compared with the asymmetric-motor group (*F*_2,35_ = 3.64, *p*=0.305), no other statistically significant differences in baseline demographic information between the anxiety and remaining subgroups emerged.	(i) PD patients with confirmed FOG participated in the study (ii) Anxious subgroup (a) Mean H&Y stage: 2.8 ± 0.7 (b) Mean UPDRS-III ON: 38.7 ± 10.1 (c) Mean UPDRS-III OFF: 44.9 ± 9.6 (iii) Asymmetric-motor subgroup (a) Mean H&Y stage: 2.5 ± 0.9 (b) Mean UPDRS-III ON: 30.9 ± 16.6 (c) Mean UPDRS-III OFF: 34.6 ± 11.9 (iv) Sensory attention subgroup (a) Mean H&Y Stage: 2.7 ± 0.4 (b) Mean UPDRS-III ON: 37.7 ± 12.0 (c) Mean UPDRS-III OFF: 43.1 ± 8.7	(i) PAS used to assess anxiety (ii) Anxious subgroup (a) Mean PAS: 15.5 ± 6.8 (iii) Asymmetric-motor subgroup (a) Mean PAS: 9.9 (iv) Sensory attention subgroup (a) Mean PAS: 13.2 ± 9.6	(i) FOG was assessed with the C-FOG as well as the FOG-Q total (ii) Each participant's gait was assessed over the course of 8 walking trials, both ON and OFF dopaminergic medication, with testing order counterbalanced across patients. The percentage of freezing time (%FOG) was also calculated. (iii) Anxious subgroup (a) Mean FOG total: 11.7 ± 3.0 min (vi) Asymmetric-motor subgroup (a) Mean FOG total: 10.7 ± 3.3 min (v) Sensory attention subgroup (a) Mean FOG total: 11.8 ± 5.3 min (vi) %FOG was significantly positively related to the anxiety subgroup data cluster (*r* = 0.43)—this result formed the basis of delineating an anxiety subgroup	(i) Mean PAS scores were significantly higher in the anxious subgroup compared to the asymmetric-motor (*t*_26_ = −2.19, *p*=0.038) and sensory attention (13.2 ± 9.6, *t*_26_ = −1.81, *p*=0.082) subgroups (ii) Significantly higher degrees of freezing on anxiety-related items of the C-FOG compared to the motor-related (*t*_14_ = −3.91, *p*=0.002) and set shifting- related items (*t*_14_ = 4.34, *p*=0.001) within the anxious subgroup

Lagravinese et al. [43]	(i) An experimental study to examine the effect of emotional stimuli on gait in PD patients with and without FOG (ii) Baseline assessment data, including anxiety outcome measures, performed on all participants (iii) Participants were recruited from the University of Genova's Department of Neuroscience	(i) Italian population (ii) 30 patients with PD (iii) PD FOG+ (*n* = 15) (a) No gender demographics (b) Mean age: 71.87 ± 4.75 (c) Mean PD duration: 11.20 ± 5.22 years (iv) PD FOG− (*n* = 15) (a) No gender demographics (b) Mean age: 73 ± 6.31 (c) Mean PD duration: 10.42 ± 5.34 years (v) 14 healthy age-matched controls (ELD) (a) No gender demographics (b) Mean age: 66.58 ± 6 (vi) Inclusion criteria: an H&Y stage of ≤3 and ability to walk unassisted (vii) Exclusion criteria: MMSE <24, history of additional neurological disorders, and any visual/vestibular/orthopaedic impairment that would negatively affect task performance (viii) There were no statistically significant differences between groups for age (*p*=0.15), disease duration (*p*=0.56), H&Y stage (*p*=0.15), or UPDRS-III score (*p*=0.23).	(i) A diagnosis of idiopathic PD made by a trained neurologist based on UK Brain Bank Criteria (ii) PD FOG+ (a) Mean H&Y stage: 2.43 ± 0.58 (b) Mean UPDRS-III: 27.18 ± 13.13 (iii) PD FOG− (a) Mean H&Y stage: 2.05 ± 0.64 (b) Mean UPDRS-III: 20.37 ± 10.27	(i) BAI used to assess anxiety (ii) PD FOG+ (a) Mean BAI: 9.35 ± 4.82 (iii) PD FOG− (a) Mean BAI: 9.87 ± 7.41 (iv) ELD Mean BAI: 5.42 ± 3.73	(i) The nFOG was used to delineate the PD FOG+ and PD FOG− groups, as well as a measure of FOG severity in the PD FOG+ group (ii) PD FOG+ (a) nFOG-Q total: 15.28 ± 4.50	(i) No statistically significant differences in BAI scores between PD FOG+, PD FOG−, and ELD groups (*p*=0.08)

Stefanova et al. [41]	(i) An experimental study performed in to explore whether attentional set shifting and inhibitory control were associated with FOG in PD (ii) Baseline assessment data, including anxiety outcome measures, performed on all participants (iii) Participants were recruited from the University of Belgrade's Institute of Neurology	(i) Serbian population (ii) 66 patients with PD (iii) Freezers (*n* = 30) (a) No gender demographics (b) Mean age: 64.90 ± 8.29 (c) Mean PD duration: 11.61 ± 4.42 years (iv) Nonfreezers (*n* = 36) (a) No gender demographics (b) Mean age: 64.67 ± 7.16 (c) Mean PD duration: 10.09 ± 5.94 (v) Healthy age and sex-matched controls (*n* = 22) (a) No gender demographics (b) Mean age: 65.04 ± 7.64 (vi) Inclusion criteria: ≥ 45 years old, an H&Y stage of <4, stable and optimized on antiparkinsonism treatment during the 4 weeks prior to study initiation and an MMSE ≥25 (vii) Exclusion criteria: significant comorbidities limiting gait, major depression according to DSM-IV criteria, and treatment with anticholinergic medications	(i) A diagnosis of idiopathic PD made by a trained neurologist based on UK Brain Bank Criteria (ii) Freezers (a) Mean H&Y stage: 2.77 ± 0.50 (b) Mean UPDRS-III: 37.50 ± 11.44 (iii) Nonfreezers (a) Mean H&Y stage: 2.29 ± 0.64 (b) Mean UPDRS-III: 32.33 ± 9.84	(i) HAMA used to assess anxiety (ii) Freezers (a) Mean HAMA: 10.07 ± 6.10 (iii) Nonfreezers (a) Mean HAMA: 8.18 ± 6.10 (iv) Control group Mean HAMA: 6.45 ± 4.41	(i) A score of ≥1 on FOG-Q3 used to separate freezers and nonfreezers (ii) Two of the following criteria required for FOG confirmation (a) Direct observation of FOG by 2 experienced neurologists (b) Self-reported FOG by the participant (c) Recognition of past episodes of FOG occurrence when the phenomena was described by a physician (iii) Mean FOG-Q total score (a) Freezers: 12.85 ± 4.88 (b) Nonfreezers: 6.28 ± 5.50	(i) HAMA differences between the groups were not statistically significant (*p*=0.182)

Raffo De Ferrari et al. [15]	(i) An experimental study examining the relationship between Theory of Mind and FOG presence in PD patients (ii) Baseline assessment data, including anxiety outcome measures, performed on all participants (iii) Participants recruited from the University of Genoa's Department of Neuroscience	(i) Italian population (ii) 29 patients with PD (iii) PD FOG+ (*n* = 15) (a) No gender demographics (b) Mean age: 72 ± 4.5 (c) Mean PD duration: 10.2 ± 6.3 years (iv) PD FOG− (*n* = 14) (a) No gender demographics (b) Mean age: 71 ± 4.3 (c) Mean PD duration: 7.9 ± 3.5 years (v) Healthy age-matched controls (*n* = 19) (a) No gender demographics (b) Mean age: 71 ± 6.8 (vi) Inclusion criteria: a H&Y stage ≤3, and an MMSE >24 (vii) PD group exclusion criteria: met DSM-IV criteria for major depression or were undergoing treatment via deep brain stimulation (viii) Healthy control exclusion criteria: a diagnosis of PD or other neurological disorder, major depression, or an MMSE <24 (ix) There were no statistically significant differences present between age or gender between groups (*p* always >0.05)	(i) A diagnosis of idiopathic PD made by a trained neurologist based on UK Brain Bank Criteria (ii) PD FOG+ (a) Mean H&Y stage: 2.6 ± 0.5 (b) Mean UPDRS-III: 31 ± 9.5 s (iii) PD FOG− (a) Mean H&Y stage: 2.4 ± 0.5 (b) Mean UPDRS-III: 30.8 ± 9.5	(i) BAI used to assess anxiety PD FOG+ (a) Mean BAI: 11.9 ± 10.7 (ii) PD FOG− (a) Mean BAI: 7.5 ± 6.8 (iii) Control group Mean BAI: 7.8 ± 6.4	(i) nFOG was used to separate PD participants into freezers and nonfreezers (ii) FOG presence confirmed via direct observation by a movement disorders neurologist in clinic using 360° turning tasks (iii) PD FOG+ (a) nFOG-Q total: 11.3 ± 3.9	(i) No significant main effect for group was found for the BAI (*F*(2.47 = 1.39, *p*=0.25)

Vandenbossche et al. [30]	(i) An experimental study investigating executive functioning and attention in PD patients with FOG with participants assessed in their home environment (ii) Baseline assessment data, including anxiety outcome measures, performed on all participants	(i) Belgian population (ii) 22 patients with PD (iii) Freezers (*n* = 11) (a) 9 males; 2 females (b) Mean age: 67.32 ± 2.72 (c) Mean PD duration: 10.45 ± 0.70 years (iv) Nonfreezers (*n* = 11) (a) 9 males; 2 females (b) Mean age: 68.20 ± 1.74 (c) Mean PD duration: 8.55 ± 1.09 years (v) Healthy age-, gender-, and education-matched controls (*n* = 10) (a) 8 males; 2 females (b) Mean age: 66.10 ± 2.27 (vi) Inclusion criteria: no additional neurological or orthopaedic disorders (vii) Exclusion criteria: MMSE <24 (viii) No significant differences were present between age and gender, with *p* values consistently >0.45	(i) Confirmed diagnosis of idiopathic PD by a specialist neurologist (who administered both neurological and neuropsychological examinations) (ii) Freezers (a) Mean H&Y stage: 2.5 ± 0.39 (b) Mean UPDRS-III: 38.91 ± 3.08 (iii) Nonfreezers (a) Mean H&Y stage: 2.23 ± 0.26 (b) Mean UPDRS-III: 34.18 ± 2.46	(i) HADS-A used to assess anxiety (ii) Mean HADS-A scores (a) Freezers: 7.36 ± 0.90 (b) Nonfreezers: 6.09 ± 1.16 (c) Healthy controls: 5.50 ± 1.63	(i) nFOG used to separate freezers and nonfreezers with scores >0 meeting criteria for the freezer group	(i) Group-to-group comparisons of mean HADS-A scores were not statistically significant (*p* values consistently >0.33) (ii) A correlational analysis revealed HADS-A score was significantly associated with nFOG-Q total score (Spearman *ρ* = 0.81, *p*=0.01)

Heremans et al. [40]	(i) An experimental study exploring handwriting differences in PD patients with FOG (ii) Baseline assessment data, including anxiety outcome measures, performed on all participants	(i) Belgian population (ii) 30 patients with PD (iii) Freezers (*n* = 15) (a) 12 males; 3 females (b) Mean age: 65 ± 9 (c) Mean PD duration: 9 ± 5 years (iv) Nonfreezers (*n* = 15) (a) 10 males; 5 females (b) Mean age: 65 ± 9 (c) Mean PD duration: 8 ± 5 years (v) Healthy age-matched controls (*n* = 15) (a) 5 males; 10 females (b) Mean age: 64.3 ± 15 (vi) Inclusion criteria: between H&Y stages 1–3 during the ON state, experiencing writing problems as indexed by an UPDRS-II item 2.7 score of >1 and had no additional neurological disorders or history of depression (vii) Exclusion criteria: MMSE ≤24, disease or surgery involving the upper limb, deep brain stimulation, and vision problems which impacted performance on the writing tasks	(i) A diagnosis of idiopathic PD made by a trained neurologist based on UK Brain Bank Criteria (ii) Freezers (a) Mean H&Y stage: 2 ± 0 (b) Mean UPDRS-III: 40 ± 16 (iii) Nonfreezers (a) Mean H&Y stage: 2 ± 0 (b) Mean UPDRS-III: 29 ± 9	(i) HADS-A was used to assess anxiety (ii) Mean HADS-A scores (a) Freezers: 7 ± 4 (b) Nonfreezers: 4 ± 4	(i) A nFOG-Q score of ≥1 was used to delineate the freezer and nonfreezer groups within PD patients (ii) Mean FOG-Q total scores (a) Freezers: 14 ± 8 (b) Nonfreezers: 0 ± 0	(i) Higher mean HADS-A score evidenced by the freezers compared to the nonfreezers was not statistically significant (*p*=0.06)

Kostić et al. [31]	(i) An experimental MRI study exploring patterns of grey matter tissue loss in PD patients with FOG (ii) Baseline assessment data, including anxiety outcome measures, performed on all participants (iii) Participants were recruited from outpatient clinics in the University of Belgrade's Department of Neurology	(i) Serbian population (ii) 37 patients with PD (iii) PD-FOG (*n* = 17) (a) 10 males; 7 females (b) Mean age: 64 ± 8 (c) Mean PD duration: 12 ± 5 years (iv) PD-noFOG− (*n* = 20) (a) 12 males; 8 females (b) Mean age: 63 ± 6 (c) Mean PD duration: 11 ± 4 years (v) Healthy controls (*n* = 34) (a) 20 males; 14 females (b) Mean age: 64 ± 7 (v) Inclusion criteria: ≥ 45 years old, had an H&Y stage of <4 during the OFF state, had undergone stable and optimized antiparkinsonism treatment during the 4 weeks prior to study initiation, and had an MMSE ≥25 (vi) Exclusion criteria: significant comorbidities limiting gait, major depression according to DSM-IV criteria, treatment with anticholinergic medications, and underlying focal or diffuse brain damage (vii) No significant differences in age (*p*=0.75), gender (*p*=0.99), and disease duration (*p*=0.23) between groups	(i) A diagnosis of idiopathic PD made by a trained neurologist based on UK Brain Bank Criteria (ii) PD-FOG (a) Mean H&Y stage: 2.7 ± 0.5 (b) Mean UPDRS-III: 39 ± 12 (iii) PD-noFOG (a) Mean H&Y stage: 2.4 ± 0.6 (b) Mean UPDRS-III: 29 ± 12	(i) HAMA was used to assess anxiety (ii) Mean HAMA scores (a) PD-FOGs: 14 ± 11 (b) PD-noFOG: 7 ± 6 (c) Healthy controls: 6 ± 4	(i) A score of ≥1 on FOG-Q3 used to separate freezers and nonfreezers (ii) Two of the following criteria required for FOG confirmation (a) Direct observation of FOG by 2 experienced neurologists (b) Self-reported FOG by the participant (c) Recognition of past episodes of FOG occurrence when the phenomena was described by a physician (i) FOG-Q total scores were quantified (a) Mean FOG-Q total scores (b) PD-FOG: 16 ± 3 (c) PD-noFOG: 5 ± 3	(i) Mean HAMA scores were higher in PD-FOG compared to PD-noFOG (*p*=0.02) and healthy controls (*p*=0.002)

Vanden bossche et al. [39]	(i) An experimental study that investigated whether sequence learning was diminished in PD patients with FOG, with participants completing a serial reaction time task with random or sequenced block conditions (ii) Baseline assessment data, including anxiety outcome measures, performed on all participants	(i) Belgian population (ii) 28 patients with PD (iii) Freezers (*n* = 14) (a) 11 males; 3 females (b) Mean age: 65.72 ± 7.91 (c) Mean PD duration: 10.21 ± 2.97 years (iv) Nonfreezers (*n* = 14) (a) 11 males; 3 females (b) Mean age: 68.03 ± 5.11 (c) Mean PD duration: 8.21 ± 3.40 years (v) Healthy age-, gender-, and education-matched controls (*n* = 14) (a) 11 males; 3 females (b) Mean age: 67.07 ± 6.64 (vi) Inclusion criteria: no additional neurological or orthopaedic disorders and normal or corrected-to-normal vision (vii) Exclusion criteria: MMSE <24	(i) Confirmed diagnosis of idiopathic PD by a specialist neurologist (who administered both neurological and neuropsychological examinations) (ii) Freezers (a) Mean H&Y stage: 2.43 ± 0.416 (b) Mean UPDRS-III: 37.21 ± 15.99 (iii) Nonfreezers (a) Mean H&Y stage: 2.43 ± 0.319 (b) Mean UPDRS-III: 35.64 ± 9.10	(i) HADS-A used to assess anxiety (ii) Mean HADS-A scores (a) Freezers: 6.57 ± 3.13 (b) Nonfreezers: 5.64 ± 3.56 (c) Healthy controls: 5.29 ± 5.54	(i) nFOG used to separate freezers and nonfreezers with scores >0 meeting criteria for the freezer group	(i) HADS-A scores were not significantly different between groups (*p*=0.693, *p*=0.019)

Rubino et al. [38]	(i) An experimental MRI study examining differences in grey matter volume between PD patients with and without FOG (ii) Baseline assessment data, including anxiety outcome measures, performed on all participants	(i) Italian population (ii) 26 patients with PD (iii) FOG+ (*n* = 13) (a) 10 males; 3 females (b) Mean age: 68.077 ± 8.221 (c) Mean PD duration: 8.154 ± 4.180 years (iv) FOG− (*n* = 13) (a) 9 males; 4 females (b) Mean age: 68.692 ± 7.664 (c) Mean PD duration: 7.077 ± 2.985 years (v) Inclusion criteria: treatment with stable dopaminergic medications, had appropriate visual acuity and hearing for the testing procedure, an MMSE >26, absence of dementia as per Movement Disorder Society clinical criteria, and no contraindications to MRI scan (vi) Exclusion criteria: additional neurological disorders, uncertain history of chronic dopaminergic medication responsiveness, unstable medical illness, history of alcoholism/drug abuse/head trauma/mental disorders, and presence of brain lesions, tumour, or marked cortical atrophy MRI (vii) No significant differences in gender, age, and disease duration between groups (*p* values consistently > 0.42)	(i) A diagnosis of idiopathic PD made by a trained neurologist based on UK Brain Bank Criteria (ii) FOG+ (a) Mean H&Y stage: 2.423 ± 0.400 (b) Mean UPDRS-III: 17.846 ± 6.22 (iii) FOG− (a) Mean H&Y stage: 2.307 ± 0.325 (b) Mean UPDRS-III: 17.230 ± 2.77	(i) HAMA used to quantify anxiety (ii) Mean HAMA scores (a) FOG+: 6.154 ± 3.95 (b) FOG−: 6.385 ± 4.46	(i) Participants were classified as FOG+ if they had a score of ≥1 on the FOG-Q3 and direct observation of FOG by 2 experienced neurologists or self-report of FOG by participant and/or the recognition of past episodes of FOG when the phenomena was described by a physician	(i) Mean HAMA scores were not significantly different in FOG+ vs. FOG− (*p*=0.890)

Pimenta et al. [33]	(i) An observational cross-sectional study examining the relationship between anxiety and FOG in PD (ii) Several self-report measures were administered to PD patients with and without FOG, including anxiety outcome measures (iii) Participants were recruited from a movement disorders clinic the State of Bahia Health Attention for the Elderly in Brazil	(i) Brazilian population (ii) 78 patients with PD (iii) Freezers (*n* = 27) (a) 16 males; 11 females (b) Median age: 71 (61–85) (c) Median PD duration: 9.0 (2–20) (iv) Nonfreezers (*n* = 51) (a) 26 males; 25 females (b) Median age: 68 (60–89) (c) Median PD duration: 4.0 (1–14) (v) Inclusion criteria: able to walk without the assistance of another person (with or without a gait aid) (vi) Exclusion criteria: atypical parkinsonism, dementia, severe visual or auditory disturbance, and presence of comorbidities that affected balance and gait. (vii)Freezers were significantly older than nonfreezers (*p*=0.010), had significantly longer disease durations than nonfreezers (*p* < 0.001), and the gender composition of each group was not significantly different (*p*=0.485) (data are shown as median (range))	(i) A diagnosis of idiopathic PD made by a trained neurologist based on UK Brain Bank Criteria (ii) Freezers (a) Median H&Y stage: 3.0 (2–4) (b) Mean UPDRS-III: 39.8 ± 9.9 (ii) Nonfreezers (a) Median H&Y stage: 2.5 (1.5–3) (b) Mean UPDRS-III: 28.4 ± 11.7	(i) Anxiety subscale of HADS (HADS-A) subtotalled and compared between groups with scores ≥ 8 meeting criteria for anxiety disorder (ii) Freezers (a) Number of PD patients with HADS-A ≥ 8: 19 (70% of freezers) (iii) Nonfreezers (a) Number of PD patients with HADS-A ≥ 8: 16 (31% of nonfreezers)	(i) Freezers were classified based on a FOG-Q3 score of ≥1 (ii) FOG items 3–6 score used to assess FOG severity	(i) Significantly higher proportions of freezers had a HADS-A score ≥ 8 than nonfreezers (*p*=0.001) (ii) Correlation analysis demonstrated a significant relationship between HADS-A and FOG-Q3 (rho = 0.44, *p* < 0.001) and FOG severity (rho = 0.36, *p*=0.001) (iii) Univariate regression analysis showed a significant association between HADS-A and FOG severity (*B* = 2.92, 95% CI: 1.24–4.59, *p*=0.001) (iv) Multivariate linear regression analysis also indicated a significant relationship between HADS-A ≥8 and FOG severity (*B* = 1.89, 95% CI: 0.47–3.31, adjusted *R*^2^ = 0.385, *p*=0.010) with a HADS-A score of ≥8 accounting for 38% of the variance in FOG severity scores
